# Towards stratified treatment of JIA: machine learning identifies subtypes in response to methotrexate from four UK cohorts

**DOI:** 10.1016/j.ebiom.2023.104946

**Published:** 2024-01-08

**Authors:** Stephanie J.W. Shoop-Worrall, Saskia Lawson-Tovey, Lucy R. Wedderburn, Kimme L. Hyrich, Nophar Geifman, CLUSTER Consortium, Aline Kimonyo, Alyssia McNeece, Andrew Dick, Andrew Morris, Annie Yarwood, Athimalaipet Ramanan, Bethany R. Jebson, Chris Wallace, Daniela Dastros-Pitei, Damian Tarasek, Elizabeth Ralph, Emil Carlsson, Emily Robinson, Emma Sumner, Fatema Merali, Fatjon Dekaj, Helen Neale, Hussein Al-Mossawi, Jacqui Roberts, Jenna F. Gritzfeld, Joanna Fairlie, John Bowes, John Ioannou, Kimme L. Hyrich, Lucy R. Wedderburn, Melissa Kartawinata, Melissa Tordoff, Michael Barnes, Michael W. Beresford, Michael Stadler, Nophar Geifman, Paul Martin, Rami Kallala, Sandra Ng, Samantha Smith, Sarah Clarke, Saskia Lawson-Tovey, Soumya Raychaudhuri, Stephanie J.W. Shoop-Worrall, Stephen Eyre, Sumanta Mukherjee, Teresa Duerr, Thierry Sornasse, Vasiliki Alexiou, Victoria J. Burton, Wei-Yu Lin, Wendy Thomson, Zoe Wanstall

**Affiliations:** a Centre for Epidemiology Versus Arthritis, The University of Manchester, UK; b Centre for Health Informatics, The University of Manchester, UK; c Centre for Genetics and Genomics Versus Arthritis, The University of Manchester, UK; d Centre for Adolescent Rheumatology Versus Arthritis at UCL, UCLH and GOSH, London, UK; e Infection, Immunity and Inflammation Research & Teaching Department, UCL GOS Institute of Child Health, London, UK; f NIHR Great Ormond Street Hospital Biomedical Research Centre, London, UK; g National Institute for Health Research Manchester Biomedical Research Centre, Manchester University Hospitals NHS Foundation Trust, Manchester, UK; h Faculty of Health and Medical Sciences, School of Health Sciences, The University of Surrey, Surrey, UK

**Keywords:** Juvenile idiopathic arthritis, Machine learning, Treatment outcome, Epidemiology, Methotrexate

## Abstract

**Background:**

Methotrexate (MTX) is the gold-standard first-line disease-modifying anti-rheumatic drug for juvenile idiopathic arthritis (JIA), despite only being either effective or tolerated in half of children and young people (CYP). To facilitate stratified treatment of early JIA, novel methods in machine learning were used to i) identify clusters with distinct disease patterns following MTX initiation; ii) predict cluster membership; and iii) compare clusters to existing treatment response measures.

**Methods:**

Discovery and verification cohorts included CYP who first initiated MTX before January 2018 in one of four UK multicentre prospective cohorts of JIA within the CLUSTER consortium. JADAS components (active joint count, physician (PGA) and parental (PGE) global assessments, ESR) were recorded at MTX start and over the following year.

Clusters of MTX ‘response’ were uncovered using multivariate group-based trajectory modelling separately in discovery and verification cohorts. Clusters were compared descriptively to ACR Pedi 30/90 scores, and multivariate logistic regression models predicted cluster-group assignment.

**Findings:**

The discovery cohorts included 657 CYP and verification cohorts 1241 CYP. Six clusters were identified: Fast improvers (11%), Slow Improvers (16%), Improve-Relapse (7%), Persistent Disease (44%), Persistent PGA (8%) and Persistent PGE (13%), the latter two characterised by improvement in all features except one. Factors associated with clusters included ethnicity, ILAR category, age, PGE, and ESR scores at MTX start, with predictive model area under the curve values of 0.65–0.71. Singular ACR Pedi 30/90 scores at 6 and 12 months could not capture speeds of improvement, relapsing courses or diverging disease patterns.

**Interpretation:**

Six distinct patterns following initiation of MTX have been identified using methods in artificial intelligence. These clusters demonstrate the limitations in traditional yes/no treatment response assessment (e.g., ACRPedi30) and can form the basis of a stratified medicine programme in early JIA.

**Funding:**

10.13039/501100000265Medical Research Council, 10.13039/501100012041Versus Arthritis, 10.13039/501100001279Great Ormond Street Hospital Children's Charity, Olivia’s Vision, and the 10.13039/501100000272National Institute for Health Research.

**Research in context**

**Evidence before this study**

Methotrexate is the gold-standard first-line disease-modifying anti-rheumatic drug (DMARD) for JIA, despite only being effective or tolerated in approximately 50% of children and young people with this disease. Stratified treatment approaches would enable those who would benefit most from methotrexate to initiate this therapy whilst re-directing others to alternative therapies. At present, in the majority of clinical trials the ‘benefits’ of therapy are measured using binary response/non-response composite outcomes, which do not account for the fact that the varied features of disease may respond differently to treatment, and that response in itself could have greater heterogeneity. Tailoring treatments based on current understanding of ‘response’ may lead to children and young people being advised to start therapies which may not benefit their specific manifestations of disease, or result in an effective therapy for some aspects of disease being discontinued. Novel methods in machine learning may be able to identify clusters of disease that have different patterns of response across features of JIA. This better characterisation of response can then facilitate more precise research into the identification of response predictors, such as biomarkers, and lead to better forecasting of likely outcomes following drug initiation. We searched MEDLINE and Embase from April 1, 1974 to Jan 1, 2020, for studies published on JIA (MeSH ‘juvenile arthritis’) on methotrexate (MeSH ‘methotrexate’) using search strings (MeSH ‘machine learning’ or ‘artificial intelligence’ or key word ‘trajectory’). While studies have predicted binary response/non-response to MTX and there is evidence for JIA disease trajectories following diagnosis, we did not find studies that explored trajectories of disease activity or impact following methotrexate therapy.

**Added value of this study**

Our work reports verifiable distinct and heterogenous clusters of JIA in terms of response of individual aspects of JIA activity and impact following methotrexate, including whether features respond in parallel or not, and the speed at which these improve. This work builds on existing studies of methotrexate treatment response, confirming that response is not bivariate but can be highly variable across different features of disease within individuals. In particular, this study confirms that one in eight children and young people starting methotrexate will demonstrate improvements in inflammatory features of disease (e.g., active joint count) yet have residual symptoms, as measured through the patient global assessment scale.

**Implications of all the available evidence**

Even where disease activity appears similar between children and young people when measured using composite disease activity scores, differences in response across measurable impacts of JIA are consistently evident. A particular key finding of this study is the verified pattern of fast versus slow response to MTX, showing that in some children, improvements in disease activity can be slower than in others over time. These different speeds of response over time are not identifiable with traditional binary treatment response measures. The reasons for these different speeds of response require further investigation to understand if this is a true biological observation, or whether it is a marker for other aspects of treatment, such as concurrent therapies (e.g., glucocorticoids), or reflect other aspects of medicines, such as adherence. Using a bivariate response definition at an earlier set point in time may misclassify some children, who later respond, as non-responders. The longer-term impact of this slower disease control needs further investigation. Our study also demonstrates the utility of machine learning methods to uncover clusters of children as a basis for stratified treatment decisions.

## Introduction

The gold standard, first-line disease-modifying anti-rheumatic drug (DMARD) for children and young people (CYP) with juvenile idiopathic arthritis (JIA) is methotrexate (MTX). This drug has proven effective at controlling disease activity, including reducing the number of active joints, as well as reducing pain and improving quality of life across JIA categories.^1^ Nevertheless, MTX is not effective for every CYP with JIA, with between 30 and 70% achieving a clinical response following its initiation.^2^, ^3^, ^4^ Others may have to stop the drug due to intolerance, in particular gastrointestinal adverse effects.^5^ Even where clinical signs of inflammatory disease have been controlled, such as active joint count (AJC), persistent symptoms including pain affect around 1 in 5 CYP who reach ‘remission’.^6^

With the advent of biological therapies, there have never been more treatment options to control JIA disease activity. However, the perceived ‘window of opportunity’ for treating early JIA based on a similar phenomenon in rheumatoid arthritis^7^ suggests a short time period following disease onset within which disease activity may be optimally controlled.^8^^,^^9^ Thus, it is important to initiate drugs that are most likely to benefit CYP with JIA as first line if possible. At present, current guidelines suggest MTX as first-line DMARD for the majority of CYP with JIA, with biologics reserved when MTX is not effective or tolerated.^10^ For patients in whom MTX will not be effective, waiting to start a biologic has the potential to prolong disease symptoms and their impact on everyday lives of CYP and their families. It also wastes time, money and effort for healthcare services by funding treatments which will not be effective but may still result in adverse events.^11^ Currently, it is not possible to predict response to MTX.

To understand efficacy of treatments, current clinical trials in JIA measure response as a primary outcome at a designated time-point from treatment initiation, with a threshold applied to dichotomise a composite measure into response/non-response. Such measures include the American College of Rheumatology Pediatric (ACR Pedi) scores or juvenile arthritis disease activity score (JADAS) improvement cut-offs.^12^ These composite scores contain heterogeneous measures of disease, designed to capture the variable features of JIA, yet are applied by dichotomising CYP into responders and non-responders, even if there is a heterogeneous response across the components. This risks misclassification of children’s response if all ‘response’ is assumed to be the same, which can compromise studies looking to identify predictors of response, such as biomarkers. In order to facilitate precision medicine research and better characterise response across children with JIA, new methods in artificial intelligence can be utilised.

Novel unsupervised methods in artificial intelligence can identify clusters of CYP who may have different patterns across multiple disease features over time. Such methods have been utilised in adult rheumatology to demonstrate shared patterns of pain and physical function in osteoarthritis^13^ and across multiple dimensions of illness perception^14^ and fatigue^15^ in rheumatoid arthritis. Recently, six clusters of CYP, based on individual components of the JADAS, were identified in a multicentre UK cohort following a diagnosis of JIA.^16^ Each cluster had different initial levels of disease activity, with three of six groups starting with ‘low’ disease, and three with ‘high’ disease at diagnosis alongside different patterns of change across individual JIA outcome measures. This approach helped identify and incorporate patterns of disease that include: reaching remission, continuing low-level disease, persistent moderate-high disease, and disease relapse. In addition, in two groups (22% of the population), active joint counts and physician global scores improved following diagnosis, but parent global scores did not.^16^ This demonstrates that, in a heterogeneous disease such as JIA, assuming that CYP either ‘improve’ or ‘do not improve’ over time is likely an oversimplification of disease patterns.

This study used unsupervised machine learning methods to uncover clusters of JIA with different patterns of disease outcomes following MTX initiation.

## Methods

### Discovery study population

The UK JIA Biologics Register includes two cohorts: the British Society for Paediatric and Adolescent Rheumatology Etanercept Cohort Study (BSPAR-ETN) and Biologics for Children with Rheumatic Diseases (BCRD). BSPAR-ETN was established in 2004 to explore outcomes following ETN therapy and BCRD in 2010 for non-etanercept bDMARDs. Both also recruit patients starting MTX as a ‘comparison’ group. These national multicentre registers run in parallel, using the same case report forms and recruit from the same UK centres. CYP can be recruited to the studies if under the age of 16 years, within six months of initiating a biological therapy or MTX and have a rheumatologist’s diagnosis of JIA.

CYP with JIA in the MTX arm of the UK JIA Biologics Register were included in the current analysis if recruited prior to 1st January 2018, to allow for at least one year of follow-up at the time of analysis. Those with no date of MTX initiation or who initiated a biologic within a month of registration, which may have represented either rapid intolerance of MTX or a delayed recording of MTX start date, were excluded. In addition, those who appeared to be in clinically inactive disease (CID), i.e., the presence of no active joints or CID using the JADAS10^17^ at MTX initiation, were excluded from all analyses to account for MTX started for a non-articular disease aspect or data inconsistencies. CYP were also excluded if they did not have all four of the JADAS components recorded simultaneously at any single point over follow-up.

### Verification study populations

Inclusion and exclusion criteria from the primary study population were applied to select CYP from the following cohorts for model verification. Models were verified in the following cohorts separately.

The Childhood Arthritis Prospective Study (CAPS) is one of the largest prospective inception cohort of JIA globally,^18^ with over 1700 CYP recruited to date. This cohort started recruiting in 2001 and recruits from seven UK paediatric rheumatology clinics at the point of initial presentation to paediatric rheumatology. CAPS includes CYP who initiate a variety of treatments, including those prescribed MTX, who were selected for the current study.

The Childhood Arthritis Response to Medication Study (CHARMS) is a multicentre treatment register recruiting CYP with JIA at the point of starting MTX or anti-TNF therapies. Two UK centres recruit CYP prospectively and additional five UK centres recruit retrospectively. Those with prospective data collection were included in the current study.

Since all cohorts were recruited from UK paediatric rheumatology clinics, some CYP were enrolled in multiple studies. Duplicates were identified via their unique NHS numbers and CYP who were enrolled in the UK JIA Biologics Register were excluded from the verification cohorts, and those enrolled to both CAPS and CHARMS were included only in the CAPS verification analysis.

#### Ethics

Ethical approvals were gained from the Northwest Greater Manchester Central Research Ethics Committee (BCRD), West Midlands Multicentre Research Ethics Committee (BSPAR-ETN), Northwest Multicentre Ethics Committee (CAPS: REC/02/8/104, IRAS 184042) and the Bloomsbury/Central London Research Ethics Committee (CHARMS: REC 05/Q0508/95, IRAS 172219). No additional ethical permissions were required for this analysis. Written informed consent was provided by guardians of participants and age-appropriate consent/assent was provided by participants themselves, where appropriate.

### Data collection

#### Discovery study populations

Data from the UK JIA Biologics Register were collected at MTX initiation and then at approximately 6 and 12 months following this date. Prior to 2008, an additional 3-month follow-up was also included. At each follow-up, demographic, disease features and medication data were extracted from the medical record by the local rheumatology team or designated local research nurse. In addition, participants (or their guardians) were asked to complete the Childhood Health Assessment Questionnaire (CHAQ), incorporating a parent global evaluation (PGE) visual analogue scale (VAS, 100 mm) and 100 m pain VAS.

#### Verification study populations

CAPS collects data at initial presentation to paediatric rheumatology, and annually thereafter. Between 2001 and 2010, an additional six-month follow-up was undertaken. Additional follow-up data are collected at MTX initiation and six months later, particularly focusing on disease activity measures. CHARMS collects data at initiation of MTX and at an approximate six-month follow-up. At each time point for both verification studies, study nurses extract demographic and clinical data from the medical record, with participants also asked to complete the CHAQ, incorporating a 100 mm PGE and for CAPS only, a 100 mm pain VAS.

#### Dates of MTX initiation and follow-up across all cohorts

Although the four studies aimed to extract data at fixed follow-up points, date of actual data collection often deviated from these time-points as they were captured during routine clinic appointments. Therefore, exact follow-up time was determined by subtracting the date of MTX initiation from the date of follow-up. CHARMS collected two MTX initiation dates, that of decision to treat and ‘actual’ MTX start. ‘Actual’ MTX start date was used in preference with decision to treat date used if actual start date was unavailable. If date of core outcome variable collection was unavailable, then the dates of pain VAS (where returned separately) and blood test dates were used sequentially instead. These values were then rounded to the nearest month for analysis. Baseline data collected more than three months prior to MTX initiation were censored and those captured between three months prior and MTX initiation were reset as day zero (MTX initiation) for the analyses if no data were available on the exact date of MTX initiation. Follow-up was extended to 14 months to capture variable data collection at the approximate one-year follow-ups.

### Outcomes

Primary outcomes were the four components of the JADAS71 (active joint count ≤71, physician’s global assessment of disease activity (PGA), PGE, erythrocyte sedimentation rate (ESR)) in the year following MTX initiation. The JADAS71 was used in preference to other JADAS measures to use more available data collected across all four cohorts, and allow models to distinguish between potential clusters with differing high numbers of active joints.

Secondary outcomes were American College of Rheumatology Pediatric response scores for 30% and 90% response (ACR Pedi 30/ACR Pedi 90). These are defined by either 30% or 90% improvement in at least three of the six JIA core outcome variables, with no more than 30% worsening in one variable.^19^

### Statistics

#### Multivariate trajectory modelling

Multivariate group-based trajectory models (GBTM) were used to classify CYP into clusters based on shared response to MTX across multiple outcomes (the four JADAS71 components). The underlying theory behind these models has been described by Nagin et al.^20^ These models allow follow-up time points and lengths to vary between CYP, with the month of follow-up from MTX start up to 14 months used to build trajectories in the present study. Given the follow-up schedule for the cohorts, each CYP had a maximum of four follow-up points within this time window. Under GBTM, a conditional independence assumption is made at the group level, unlike random effects models in which the assumption is made at the individual level. In trade, GBTM does not assume that this random effect is independently or identically distributed across groups according to a normal distribution.^20^

Using time as the independent variable, first, the number and polynomial forms of trajectories to be produced by the model are specified. Posterior probabilities of group assignment are then produced (given the data, how likely is it for each person to belong to each of the clusters). Each CYP is assigned to the group for which they have the highest posterior probability of membership. The outcome components were modelled using censored-normal models. Each outcome was log1p transformed for analysis. Linear, quadratic and cubic polynomials were tested independently. Within each polynomial form, one to ten trajectories were tested.^21^

To select the optimal model, initially, models were excluded if they resulted with a trajectory group that included <1% of the cohort, or, with a mean posterior probability for group assignment <70%, or relative model entropy (a measure of classification accuracy) at <0.5.^21^ Optimal models were then selected based on model fit (Bayesian Information Criteria, BIC, nearest zero) and a final model selected based on clinical relevance.

#### Competing risks

These analyses sought to understand groups of CYP with different treatment response patterns following MTX initiation; however, MTX may be discontinued for a number of reasons including intolerance and/or inefficacy. At this point, the CYP may be switched to an alternative therapy, largely bDMARDs. Where CYP were switched to a bDMARD, outcome data were censored on the day after biologic initiation to avoid capturing response from an alternative therapy. Any outcome data collected on the day of initiation was included since any new therapy would not have affected these outcomes. MTX survival to biologic addition or switching were compared across trajectory groups via Kaplan–Meier statistics. Outcomes following MTX cessation where an alternative therapy was not added were retained, in order to capture the natural disease course following MTX initiation, including remission off medication.

#### Clinical characteristics of MTX response clusters

Associations between demographic, psychosocial, and clinical factors collected at MTX initiation and MTX response clusters were explored descriptively and through univariable and multivariable multinomial logistic regression analyses. All non-collinear variables were entered into multivariable models, which were tested for predictive ability using receiver operating characteristics. Where pairs of variables were collinear, that with greater available data was prioritised. Each cluster was also compared descriptively against ACR Pedi 30 and 90 criteria 6 and 12 months following MTX initiation. Proportion achieving each ACR Pedi criteria set across MTX clusters alongside 95% confidence intervals are reported.

#### Missing data

Group-based trajectory modelling is a maximum-likelihood-based technique and as such, is robust to bias occurring from missing data that are missing at random.^20^ Although no imputations of data were undertaken for the primary analysis, the differences in missing data patterns across follow-up, time to biologic switching and adverse events were assessed graphically between identified multivariate clusters to identify if group assignment was driven by missing data patterns.

To assign ACR Pedi response at six and 12 months, missing JIA core outcome variables were imputed over 20 datasets using data from baseline (month 0), six months (4–8 months) and one year (10–14 months). These analyses were undertaken in the previous set of participants that had at least one time point with complete JADAS data, but may not have had complete JADAS data at six or 12 months. Age, gender, ethnicity and ILAR category at baseline and pain at all three time points additionally contributed to the imputation model. Only those CYP with available demographic and ILAR category data were included in this analysis (n = 644/657).

#### Model verification

Model verification was undertaken by independently repeating the modelling approach taken in the discovery cohorts in the two verification cohorts. These models were built entirely separately in each cohort, not using the discovery model as a guide and not seeking to ‘match’ groups in any way beyond finding the optimally-fit, most clinically relevant model in each separate cohort. Linear-only polynomials were tested in CHARMS, since this cohort collects a maximum of two time points per participant. Since follow-up time between these time points could determine trajectory assignment, in the CHARMS verification models, length of follow-up was included as a covariate in the group-based trajectory models. Optimal models were compared between discovery and verification cohorts for number and size of clusters in addition to qualitative similarities in trajectory patterns identified.

### Role of funders

The funder of the study had no role in study design, data collection, data analysis, data interpretation, or preparation of this manuscript. The corresponding author had full access to all data in the study and had final responsibility for the decision to submit for publication.

## Results

### Study populations

**Fig. 1:**
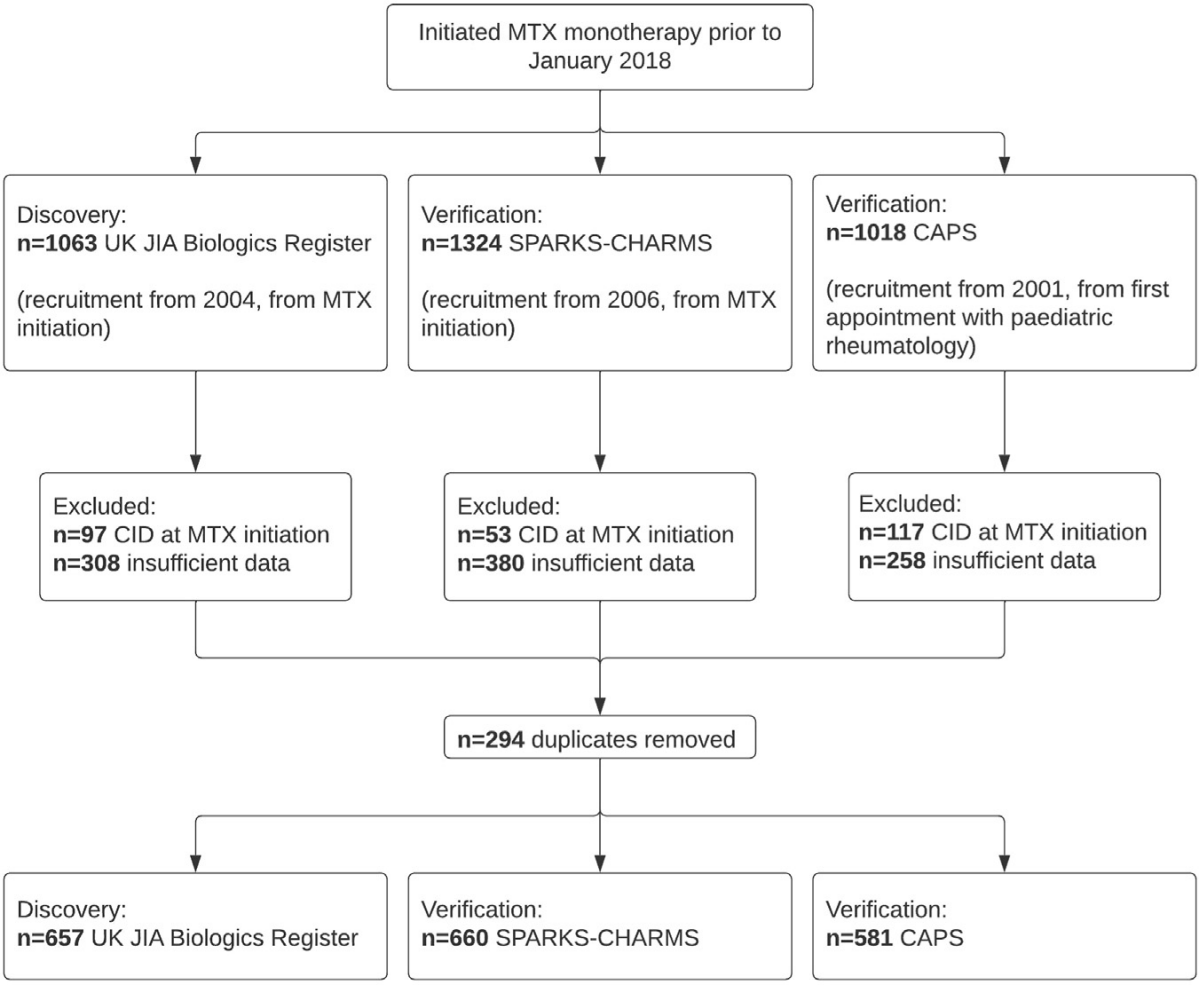
Cohort inclusion flowchart for the current study.

A total of 1898 CYP were included in the current study; 657 in the UK JIA Biologics Register discovery cohort, 581 in the CAPS verification cohort and 660 in the CHARMS verification cohort (Fig. 1). Demographic and disease features at MTX initiation were similar across all studies (Table 1). Those excluded from the discovery analysis, largely for missing data, had marginally greater representation of white ethnicities (included 84% white, excluded 91% white, p = 0.003, chi-squared test) and slight differences in ILAR representation (p = 0.032, chi-squared test) but did not differ among other clinical variables or demographics.

**Table 1:** Baseline characteristics of the discovery and verification cohorts at the points of initiating MTX.

Characteristic at MTX initiation	Discovery cohort		Verification cohorts			
	UK JIA biologics register (n = 657)		CAPS (n = 581)		CHARMS (n = 660)	
	% available	N (%) or median (IQR)	% available	N (%) or median (IQR)	% available	N (%) or median (IQR)
**Demographic**						
Female^a^	97	439 (69)	100	402 (69)	99	418 (64)
Age (yrs)	98	9.4 (4.2, 13.3)	>99	8.1 (4.1, 12.2)	98	8.3 (4.3, 11.5)
Disease duration (months)	45	6.4 (2.2, 17.6)	98	7.2 (3.6, 16.6)	94	8.9 (4.1, 25.8)
White ethnicity	97	535 (84)	>99	479 (83)	99	598 (92)
**ILAR category**						
Systemic	95	19 (3)	98	45 (8)	96	51 (8)
Oligoarthritis						
Persistent		114 (18)		114 (20)		98 (15)
Extended		114 (18)		47 (8)		134 (21)
RF− Poly		208 (33)		183 (32)		226 (36)
RF+ Poly		64 (10)		31 (5)		41 (6)
ERA		40 (6)		32 (6)		33 (5)
PsA		47 (8)		55 (10)		48 (8)
Undifferentiated		21 (3)		62 (11)		4 (1)
**Disease features**						
Active joint count, n (0–75)	95	5 (2, 10)	89	5 (3, 9)	97	6 (3, 10)
Limited joint count, n (0–75)	94	4 (2, 7)	88	3 (1, 6)	95	4 (2, 8)
Physician global (0–100 mm)	75	40 (23, 60)	55	28 (6, 52)	88	40 (26, 60)
Parent global (0–100 mm)	78	42 (20, 61)	50	33 (9, 55)	82	41 (19, 65)
CHAQ (0–3)	74	1.0 (0.4, 1.5)	43	1.0 (0.3, 1.8)	80	1.0 (0.4, 1.8)
ESR (mm/h)	91	16 (7, 37)	77	26 (11, 55)	93	29 (11, 58)
Pain (0–100 mm)	74	50 (20, 70)	41	46 (12, 78)	0	–
History of uveitis	92	53 (9)	75	26 (6)	81	105 (20)

a Gender was captured as male/female across all studies.

#### Discovery of multivariate trajectories of JADAS following MTX initiation

**Fig. 2:**
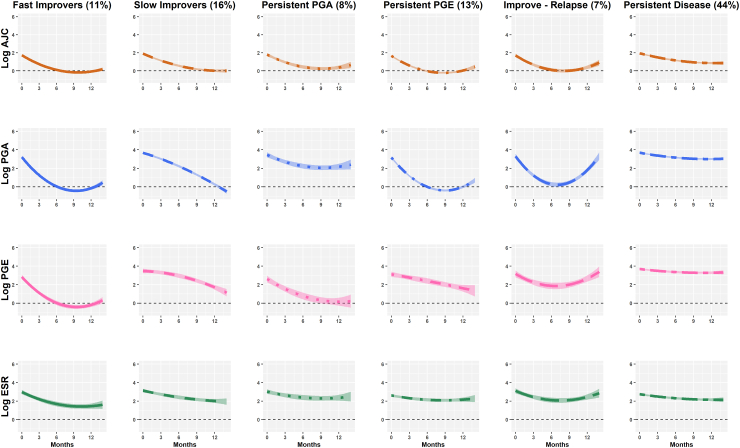
Average log1p transformed active joint count, physician global, patient/parent global and ESR trajectories within six multivariate disease clusters over the year following MTX initiation in the discovery cohort. Shaded patterns on average trajectories are purely for visualisation and comparison with verification cohort trajectories, and are not related to underlying data. AJC: active joint count; PGA: physician’s global assessment; PGE: parental global evaluation; ESR: erythrocyte sedimentation rate.

Within the discovery cohort, the optimal model classified six quadratic trajectory clusters (Fig. 2, Supplementary Figures S1 and S2 and Supplementary Table S1). ESR trajectories appeared similar between the six clusters. In two clusters, AJC, PGA and PGE scores improved in parallel at a faster (Fast Improvers: 11%) or slower (Slow Improvers: 16%) speed over the one year period. One cluster demonstrated improvement and then relapse in these three outcomes (Improve-Relapse: 7%). In two clusters, the different components followed divergent trajectories, with one cluster maintaining non-zero PGA scores despite improving AJC and PGE (Persistent PGA: 8%) and another non-zero PGE scores despite improving AJC and PGA (Persistent PGE: 13%). A final, larger, cluster maintained higher scores across all outcomes (Persistent Disease (44%).

#### ACR Pedi 30/90 achievement across multivariate trajectory groups following MTX initiation

**Fig. 3:**
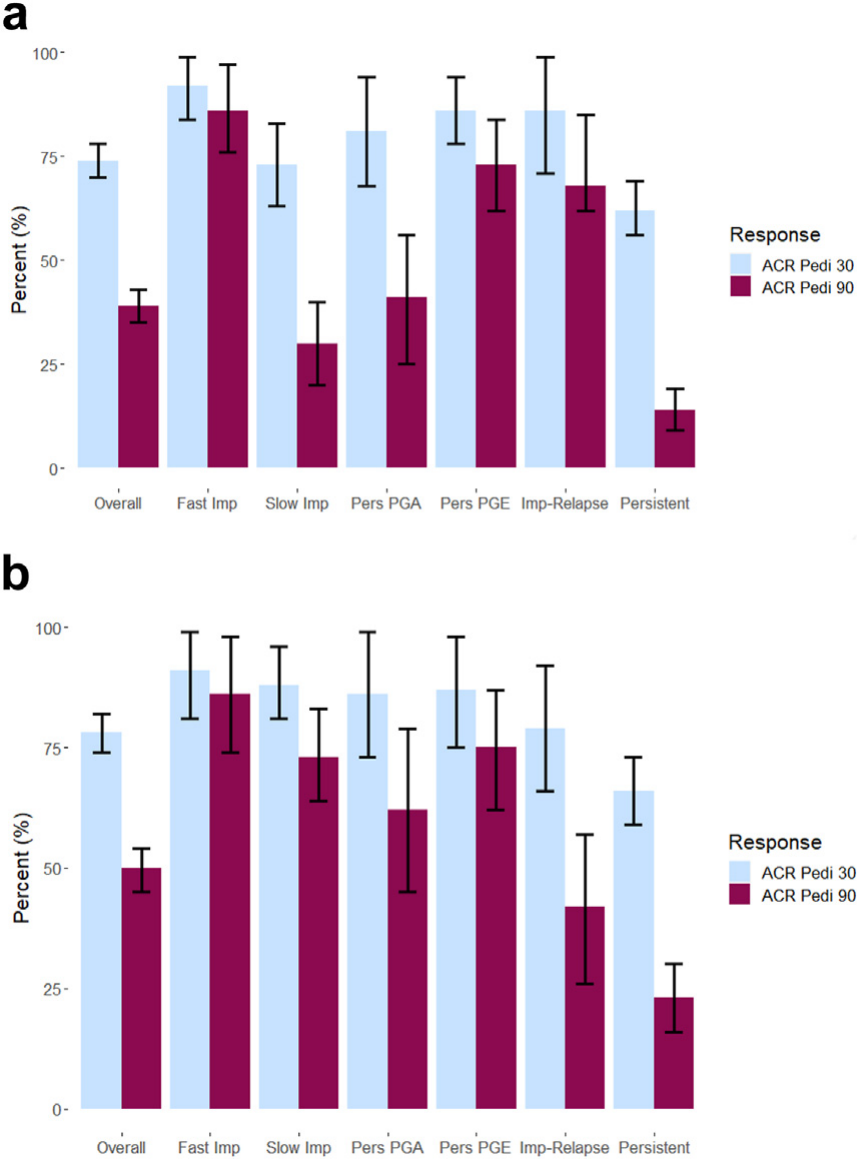
ACR Pedi 30 and 90 achievement across the 6 multivariate trajectory groups following MTX initiation in the discovery cohort: a) within 6 months, b) within 12 months. Raw numbers are presented in Supplementary Table S2.

ACR Pedi 30 and 90 achievement was met by 74% (95% CI 70, 78) and 39% (95% CI 35, 43) of CYP at six months, respectively, and 78% (95% CI 74, 82) and 50% (95% CI 45, 54) at 12 months, respectively. ACR Pedi 30 response was high across all groups, including the Persistent Disease cluster, at both 6 (range 62–92) and 12 months (range 66–91) (Fig. 3, Supplementary Table S2).

ACR Pedi 90 achievement was high at both six and 12 months in the Fast Improvers (86%, 86%) and Persistent PGE clusters (73%, 75%) and low at these time points in the Persistent Disease cluster (14%, 23%). Both the Slow Improver and Persistent PGA clusters had higher ACR Pedi 90 response at 12 (73%, 62%) months than 6 months (30%, 41%). Those in the Improve-Relapse group had higher ACR Pedi 90 response at six months (68%) than 12 months (42%) (Fig. 3, Supplementary Table S2).

#### Clinical characteristics of CYP within each multivariate trajectory group following MTX initiation

There were similar distributions of data availability over follow-up across the identified groups (Supplementary Figure S3), and time between registration and MTX start were similar across clusters with different speeds of improvement following MTX (Supplementary Figure S4). However, there were significant differences in the proportion of switching to biologics, and time to biologic therapies across the groups within the 14 months following MTX initiation, with greater (38%) and faster switching in the Persistent Disease cluster and lowest and slowest in the Fast Improvers (9%) and Persistent PGE (8%) clusters (Supplementary Table S3, Supplementary Figure S5). There was no significant difference in the proportion that had stopped MTX due to an adverse event/intolerance between clusters (Supplementary Table S3).

**Fig. 4:**
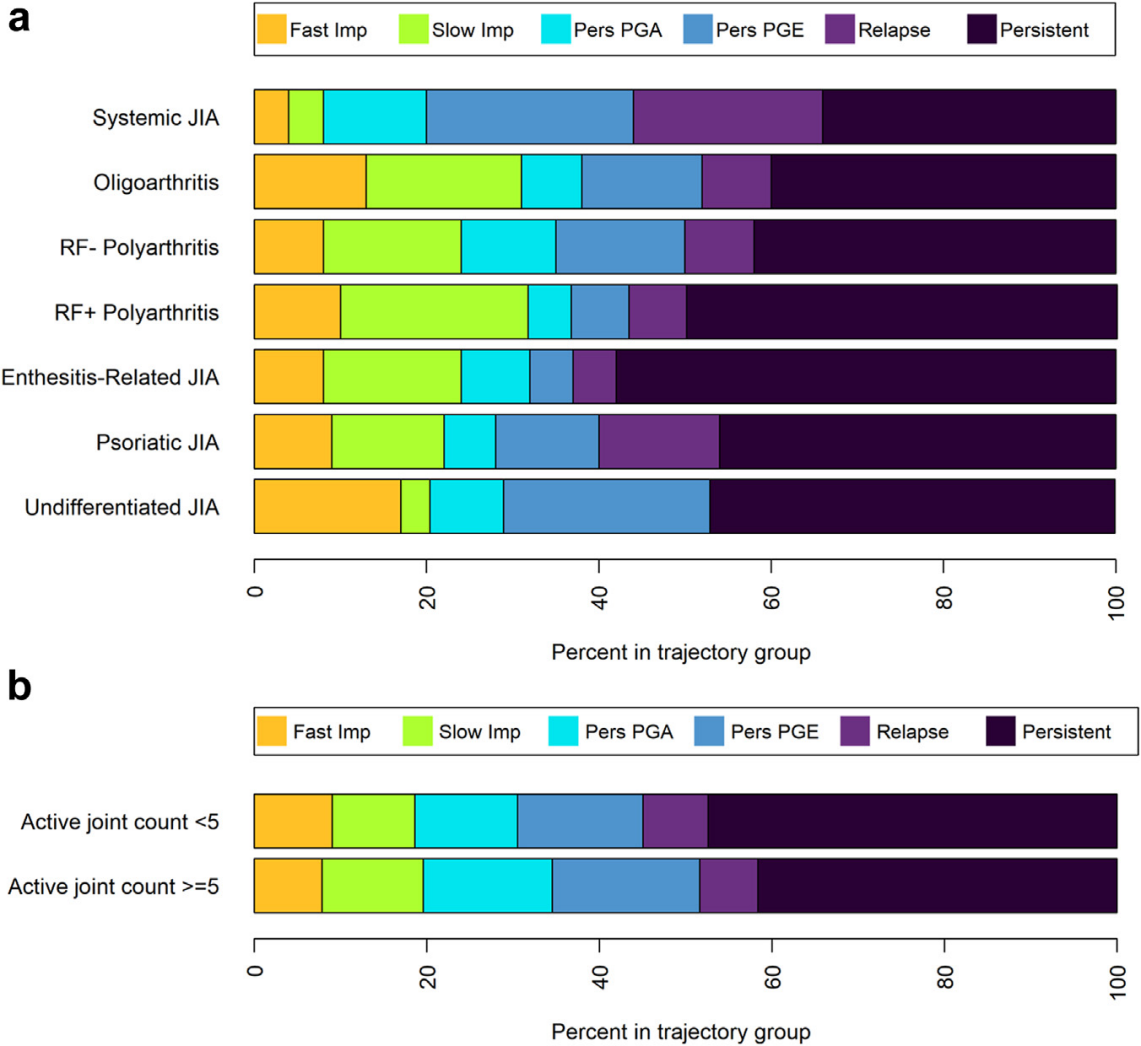
Representation of a) ILAR categories and b) oligoarticular or polyarticular joint counts at MTX initiation between the 6 multivariate trajectory clusters following MTX initiation in the discovery cohort.

Although there were statistically significant differences in baseline age and all JIA core outcome variables between clusters in univariable analyses (Fig. 4, Supplementary Table S3), few factors were independently associated with cluster assignment after multivariable adjustment. Compared with CYP in the Persistent Disease cluster, with increasing age, CYP had lower odds of being in the Fast Improvement (OR: 0.92, 95% CI 0.86, 1.00), Slow Improvement (OR: 0.89, 95% CI 0.83, 0.96) or Improve-Relapse (OR: 0.91, 95% CI 0.83, 1.00) clusters. With each increased mm of PGE, CYP had lower odds of being in the Persistent PGA cluster (OR: 0.97, 95% CI 0.96, 0.99) and with each increase in mm/h ESR, CYP had lower odds of being in the Persistent PGE cluster (OR: 0.98, 95% CI 0.97, 1.00) than the Persistent Disease cluster. Children of white ethnicities also had lower odds of being in the Persistent PGE cluster (OR: 0.46, 95% CI 0.21, 1.00) and those with enthesitis-related or undifferentiated JIA had lower odds of being in the Improve-Relapse than Persistent Disease cluster compared with those with persistent oligoarthritis (Supplementary Table S4). A multivariable model adjusting for factors in Supplementary Table S4, had area under the curve values of 0.67 for Fast Improvers, 0.71 for Slow Improvers, 0.69 for Persistent PGA, 0.65 for Persistent PGE, 0.70 for Improve-Relapse and 0.70 for Persistent Disease clusters when compared with all other clusters (Supplementary Figure S6).

#### Verification cohorts: multivariate trajectories of JADAS following MTX

All trajectory patterns identified in the discovery cohort except for the Improve-Relapse cluster were also identified in the two verification cohorts (Table 2, Supplementary Tables S5 and S6, Supplementary Figures S7–S9). In the CAPS cohort, the Persistent PGA and Persistent PGE patterns were joined in a single cluster.

**Table 2:** Percentage of cohorts within each trajectory group identified in the discovery and verification cohorts.

Cohort	Cohort use	Trajectory pattern (%)					
		Fast improvers	Slow improvers	Persistent PGA	Persistent PGE	Improve-relapse	Persistent disease
UK JIA Biologics Register	Discovery	8	11	13	16	7	44
CAPS	Verification	13	17	31 combined		NA	39
CHARMS	Verification	12	26	12	11	NA	39

## Discussion

Using data from four nationwide UK prospective cohorts of JIA, this study identified multiple unique and verifiable response patterns following MTX initiation that extend beyond the traditional response/non-response paradigm, showing variability in improvement in individual features over time. Key features of uncovered clusters include different speeds of improvement recorded over time and discordance between active joint count and/or physician or parent global scores.

Assessing treatment response is the cornerstone of clinical trials of new therapies, allowing a wider range of disease-modifying drugs for greater personalisation of JIA treatment. In such trials, treatment response has, to date, been largely assessed based on ACR Pedi scores.^12^ The high achievement of ACR Pedi 30 response across all clusters in the current study demonstrates the minimal nature of this response criteria, and ability of anti-rheumatic drugs to achieve this minimal response, even in children who have evidence of persistent inflammation. In addition, the composite nature of this tool means that changes in individual disease features, and in particular, how these relate to each other, may not be considered when reporting outcomes and developing evidence that will feed into treatment licensing for JIA. The static nature of response criteria applied to a single point in time in clinical trials also misses dynamic patterns of disease, such as the variable rates of achieving improvement in disease, or improving then relapsing. This could result in subsequent misclassification of response or under/over-estimate the response rates of a drug. Of note, clinical trials and observational studies of new therapeutic approaches (e.g., treat-to-target, early aggressive therapeutic approaches, mono versus combination therapies or drug withdrawal following response) in existing licensed therapies for JIA use a wider variety of primary outcomes. These include binary flare/non-flare and remission/non-remission outcomes^8^^,^^22^ as well as time-to- and time-in- remission.^23^, ^24^, ^25^ The use of remission and time-in-remission outcomes includes useful information on stable disease courses over time, and the clinical level of each included disease activity feature (since all have to be low/zero to fulfil the criteria). However, these also do not allow for the understanding of common heterogeneous response patterns, demonstrated in the current study, where features of disease change differentially over time in relation to each other. This key information can be gained using multivariate outcome approaches such as trajectory clustering. These outcome patterns could also be used as alternative outcomes in clinical research, both to understand disease impact following drug initiation, as well as for investigating mechanistic predictors of drug outcome, and identifying clinically useful biomarkers. These investigations have potential to feed into greater precision medicine for JIA via directing therapies based on predicted type (or trajectory) of disease or drug outcome.

Trajectories identified in the current study are different to those identified in a previous study of JIA following diagnosis.^16^ In the prior study, three clusters had low joint counts at diagnosis, and three higher joint counts. The more homogenous nature of joint counts at MTX start compared with those at diagnosis is likely reflective of selective MTX initiation in those with moderate/severe disease, or those for whom initial extreme joint activity has been partially controlled with intra-articular glucocorticoid injections. However, both studies demonstrate a cluster that improves and then relapses, alongside a large proportion of CYP whose parent global scores are persistently raised despite improvements in clinical features. In the current study, this phenomenon is verified across multiple cohorts, and has also been reported for CYP in apparent clinical ‘remission’, but who maintain raised parent global scores,^6^ for whom long-term outcome has shown to be poorer.^26^ In the prior trajectory study, PGE scores consistently mirrored functional ability and pain scores, including where divergent from joint counts and PGA scores.^6^ The current study identifies a cluster where PGA scores diverge from other outcomes, not present in the post-diagnosis trajectories. These raised PGA scores in the current study may reflect the presence of active extra-articular features or a reluctance to mark at ‘zero’ in the presence of ongoing medication. Among over 5000 CYP with JIA in a worldwide cross-sectional study who had zero active joints, one third had raised PGA scores. This discordance was associated with extra-articular features such as enthesitis, uveitis and systemic features, alongside elevated acute phase reactants, pain, morning stiffness and psychosocial health scores.^27^ The latter beyond extra-articular features are unlikely in this study due to the normal average ESR and average 0 cm PGE score for this cluster where PGA scores are raised.

Predicting remission and treatment response in JIA has been challenging, with few clinical predictors consistently associated with these outcomes.^28^ Common predictors of response to MTX from previous studies include shorter disease duration, lower joint counts, lower functional disability and wellbeing scores and biomarkers including CRP, ANA and MRP8/14, with several candidate genetic SNPs also suggested.^4^^,^^5^^,^^29^, ^30^, ^31^ However, consistency of prediction has been an issue.^28^ To effectively implement a precision medicine approach, consistent biomarkers or other predictors of outcome must be available to form the basis for treatment stratification. This study has demonstrated that heterogeneity within a single composite response measure, such as the ACR Pedi score, may be a contributing factor, where the phenotype being predicted is not homogenous across study subjects but are assumed to represent a common disease state. To minimise such misclassification of ‘response’, this study has demonstrated how expanding outcome categories to capture more homogenous groups may improve outcome prediction. Even when controlling for ILAR category, younger age was associated with better overall response to MTX in terms of AJC, PGA and PGE. This corroborates existing evidence of an association between younger age at MTX initiation and better overall response in univariable analyses.^28^ Younger age was not associated with groups where AJC and global scores diverged. In addition, discordance between physician/inflammatory markers and parent global scores at MTX initiation may be a marker for future disease course, with persistence in the raised factor an associated outcome. Further studies should explore biological markers, inclusive of genetic markers, of more homogenous response categories, such as those presented in this study.

This study benefitted from four large, independent populations of JIA to discover and verify models, all drawn from the same general UK JIA population. These rich datasets allowed for our approach to uncover previously unverified clusters of MTX response trajectories, with clinical data available at different times in the year following MTX initiation. Similar disease patterns following MTX were evident across cohorts with different study populations and data availability. Where fewer outcome data were available in the inception versus medication-focused cohorts, the models were equipped to handle such data that were likely missing-at-random or missing-completely-at-random due to cohort follow-up design.^32^ In addition, despite differences in ILAR category distribution between the cohorts, trajectory patters were verified repeatedly. This verification suggests consistent, global disease patterns across JIA, strengthened through the observation that every ILAR category had children assigned across each of the six clusters.

Limitations of the current study include the limited frequency of data collection across the discovery and verification cohorts. With more frequent data collection, even more granular patterns may have been uncovered. Specifically, one of the verification studies only collected data at two time points, essentially constraining the potential for verifying one of the trajectory patterns that improved before relapse. However, five of the six trajectory patterns were able to be verified despite these limited data. While censoring of data at medication switch allowed the understanding of disease impact patterns following MTX drug therapy, patterns later in the disease course derive from those who stayed on the drug, and therefore may appear more favourable given the switching of those who did not respond/do not tolerate MTX. Although four large cohorts were used for this study, the majority of participants were of white ethnicity. This study was able to identify that ethnic minority participants were at higher odds of persistent poor wellbeing despite improved clinical picture of disease compared with white participants. However, it lacked numbers to explore specific ethnicities. Further work should aim to understand outcomes and drivers of differing disease outcomes across CYP with JIA with different ethnicities. In addition, greater participant numbers may have allowed greater power to compare predictors of individual trajectory patterns, for example fast versus slow improvement. Further work should explore predictors between clusters with different speeds of change in disease measures following initiation of MTX, as well as better characterisation of other features of disease and intervention in these CYP, including use of glucocorticoids over the observational period as well as medication adherence, which can also affect these disease measures. It should be noted that MTX is not indicated for every child within all categories of JIA. In the UK, following glucocorticoid therapy, those with active disease and macrophage activation syndrome unresponsive to IV steroids, sacroiliitis or axial arthritis are advised to initiate biological therapies, with present guidelines suggesting a trial of MTX in all other cases. Therefore, these results do not generalise to those CYP with the above disease phenotypes. There is also movement toward biological first-line DMARDs for systemic JIA in other populations,^33^ which is consistent with the low proportion of systemic JIA in this study assigned into ‘Fast Improver’ or ‘Slow Improver’ clusters. While this study was not powered for subgroup analysis within individual ILAR categories, the large heterogeneity in cluster assignment across all ILAR categories suggests that, at least following MTX therapy, JIA category is not driving the prediction of progression across the JADAS components. Finally, the cluster models were verified in independent cohorts, but could not be statistically validated. Model validation for unsupervised learning is currently in its infancy. However, the verification across two cohorts additional to the discovery cohort demonstrates robustness of the clusters identified in real-world data and increases our confidence that these represent true disease impact patterns.

### Conclusion

Six different patterns of response were identified and verified in four UK cohorts following MTX initiation, moving beyond the traditional response versus non-response paradigm. These patterns differed in terms of changes within individual disease measures as well as their speed of change over time. The ACR Pedi Score did not differentiate well between these different groups of children. Future studies of predictors of treatment response should consider this variability in response.

## Contributors

SSW completed the formal analyses and writing–original draft. SSW, SLT, LRW, KLH, NG and the CLUSTER consortium all contributed to conceptualisation, data curation, investigation, methodology, project administration, resources, visualisation, validation and writing–review and editing. All authors read and approved the final version of the manuscript and had access to and could verify the data. KLH, NG and LRW were involved in funding acquisition and supervision.

## Data sharing statement

Information regarding applying for access to UK JIA Biologics Register, CAPS and/or CHARMS data can be found at: http://www.caps-jia.org.uk/clinicians-and-researchers/, https://sites.manchester.ac.uk/bcrdbspar/ and https://www.clusterconsortium.org.uk/2349-2/.

## Declaration of interests

The CLUSTER consortium reports grants from AbbVie and Sobi outside the submitted work, in addition to funding from Versus Arthritis (20747), the British Society for Rheumatology, Pfizer, Sparks UK (08ICH09), the Medical Research Council (MR/M004600/1), and UK Juvenile Idiopathic Arthritis Genetics Consortium for CLUSTER cohorts. KLH reports grants from BMS and Pfizer, and speaker's fees from AbbVie, outside the submitted work. All other authors declare no other competing interests.

## Appendix A Supplementary data

Supplementary Materials

Members of the CLUSTER Consortium
