# Supplementary material for: Towards stratified treatment of JIA: machine learning identifies subtypes in response to methotrexate from four UK cohorts
Source: eBioMedicine. 2024 Jan 8;100:104946. doi: 10.1016/j.ebiom.2023.104946 (PMC10792564; doi:10.1016/j.ebiom.2023.104946)
Supplement: Supplementary Materials [file mmc1.docx]

**SUPPLEMENTARY MATERIALS**

**Supplementary Table 1.** Model diagnostics for multivariate trajectory models of log1p transformed JADAS components following MTX in the discovery cohort

| Class no. | BIC | Class percentages (%) | | | | | | | | | | Average posterior probability per class  (higher is better) | | | | | | | | | | Model average posterior prob. for assigned class  (higher is better) | Relative Entropy  (close to 1 is better) | |
| --- | --- | --- | --- | --- | --- | --- | --- | --- | --- | --- | --- | --- | --- | --- | --- | --- | --- | --- | --- | --- | --- | --- | --- | --- |
|  |  | 1 | 2 | 3 | 4 | 5 | 6 | 7 | 8 | 9 | 10 | 1 | 2 | 3 | 4 | 5 | 6 | 7 | 8 | 9 | 10 |  |  |  |
| Linear | | | | | | | | | | | | | | | | | | | | | | | | |
| 1 | 9527 | 100 |  |  |  |  |  |  |  |  |  | 1.00 |  |  |  |  |  |  |  |  |  | 1.00 | 1.00 |  |
| 2 | 8907 | 62 | 38 |  |  |  |  |  |  |  |  | 0.96 | 0.93 |  |  |  |  |  |  |  |  | 0.95 | 0.92 |  |
| 3 | 8732 | 46 | 30 | 25 |  |  |  |  |  |  |  | 0.88 | 0.86 | 0.92 |  |  |  |  |  |  |  | 0.89 | 0.83 |  |
| 4 | 8698 | 43 | 25 | 23 | 9 |  |  |  |  |  |  | 0.88 | 0.81 | 0.92 | 0.87 |  |  |  |  |  |  | 0.87 | 0.79 |  |
| 5 | 8657 | 25 | 24 | 23 | 18 | 10 |  |  |  |  |  | 0.83 | 0.78 | 0.92 | 0.79 | 0.84 |  |  |  |  |  | 0.83 | 0.71 |  |
| 6 | 8621 | 25 | 23 | 21 | 19 | 10 | 3 |  |  |  |  | 0.84 | 0.78 | 0.91 | 0.79 | 0.83 | 0.91 |  |  |  |  | 0.84 | 0.71 |  |
| 7 | 8604 | 26 | 19 | 17 | 15 | 12 | 10 | 1 |  |  |  | 0.81 | 0.77 | 0.79 | 0.87 | 0.84 | 0.91 | 0.99 |  |  |  | 0.85 | 0.69 |  |
| 8 | 8575 | 23 | 19 | 16 | 12 | 10 | 9 | 5 | 5 |  |  | 0.80 | 0.76 | 0.76 | 0.86 | 0.86 | 0.81 | 0.87 | 0.83 |  |  | 0.82 | 0.66 |  |
| 9 | 8601 | 24 | 18 | 15 | 15 | 12 | 10 | 4 | 2 | <1 |  | 0.79 | 0.77 | 0.86 | 0.76 | 0.84 | 0.91 | 0.80 | 0.91 | 0.97 |  | 0.85 | 0.68 |  |
| 10 | 8612 | 26 | 16 | 15 | 13 | 10 | 10 | 5 | 4 | 2 | <1 | 0.74 | 0.84 | 0.76 | 0.83 | 0.81 | 0.91 | 0.76 | 0.78 | 0.91 | 0.97 | 0.83 | 0.65 |  |
| Quadratic | | | | | | | | | | | | | | | | | | | | | | | | |
| 1 | 9461 | 100 |  |  |  |  |  |  |  |  |  | 1.00 |  |  |  |  |  |  |  |  |  | 1.00 | 1.00 |  |
| 2 | 8850 | 60 | 40 |  |  |  |  |  |  |  |  | 0.95 | 0.94 |  |  |  |  |  |  |  |  | 0.95 | 0.92 |  |
| 3 | 8721 | 48 | 28 | 25 |  |  |  |  |  |  |  | 0.89 | 0.91 | 0.88 |  |  |  |  |  |  |  | 0.89 | 0.83 |  |
| 4 | 8607 | 47 | 23 | 17 | 12 |  |  |  |  |  |  | 0.85 | 0.88 | 0.83 | 0.89 |  |  |  |  |  |  | 0.86 | 0.78 |  |
| 5 | 8568 | 46 | 16 | 15 | 13 | 11 |  |  |  |  |  | 0.85 | 0.82 | 0.83 | 0.88 | 0.89 |  |  |  |  |  | 0.85 | 0.76 |  |
| 6 | 8532 | 44 | 16 | 13 | 11 | 8 | 7 |  |  |  |  | 0.86 | 0.83 | 0.79 | 0.90 | 0.85 | 0.88 |  |  |  |  | 0.85 | 0.75 |  |
| 7* | 8546 | 44 | 15 | 12 | 11 | 9 | 6 | 2 |  |  |  | 0.85 | 0.82 | 0.74 | 0.82 | 0.86 | 0.86 | 0.91 |  |  |  | 0.84 | 0.73 |  |
| 8* | 8502 | 23 | 21 | 17 | 13 | 8 | 8 | 8 | 2 |  |  | 0.81 | 0.77 | 0.80 | 0.83 | 0.85 | 0.88 | 0.86 | 0.83 |  |  | 0.83 | 0.69 |  |
| 9* | 8487 | 21 | 21 | 17 | 12 | 10 | 9 | 8 | 2 | 1 |  | 0.81 | 0.79 | 0.80 | 0.88 | 0.79 | 0.83 | 0.88 | 0.96 | >0.99 |  | 0.86 | 0.70 |  |
| 10* | 8492 | 23 | 19 | 16 | 13 | 9 | 8 | 6 | 4 | 2 | <1 | 0.80 | 0.82 | 0.80 | 0.82 | 0.89 | 0.87 | 0.83 | 0.86 | 0.95 | >0.99 | 0.86 | 0.70 |  |
| Cubic | | | | | | | | | | | | | | | | | | | | | | | | |
| 1 | 9460 | 100 |  |  |  |  |  |  |  |  |  | 1.00 |  |  |  |  |  |  |  |  |  | 1.00 | 1.00 |  |
| 2 | 8846 | 63 | 37 |  |  |  |  |  |  |  |  | 0.96 | 0.95 |  |  |  |  |  |  |  |  | 0.96 | 0.93 |  |
| 3 | 8724 | 46 | 28 | 26 |  |  |  |  |  |  |  | 0.90 | 0.88 | 0.93 |  |  |  |  |  |  |  | 0.90 | 0.84 |  |
| 4 | 8586 | 46 | 23 | 22 | 9 |  |  |  |  |  |  | 0.89 | 0.87 | 0.88 | 0.86 |  |  |  |  |  |  | 0.87 | 0.81 |  |
| 5 | 8562 | 45 | 21 | 14 | 10 | 10 |  |  |  |  |  | 0.87 | 0.83 | 0.86 | 0.89 | 0.91 |  |  |  |  |  | 0.87 | 0.79 |  |
| 6* | 8517 | 29 | 24 | 22 | 16 | 6 | 3 |  |  |  |  | 0.79 | 0.83 | 0.85 | 0.85 | 0.95 | 0.93 |  |  |  |  | 0.87 | 0.74 |  |
| 7* | 8510 | 43 | 16 | 11 | 11 | 10 | 7 | 2 |  |  |  | 0.87 | 0.83 | 0.84 | 0.79 | 0.88 | 0.90 | 0.84 |  |  |  | 0.85 | 0.76 |  |
| 8 | 8485 | 23 | 22 | 16 | 13 | 10 | 7 | 6 | 4 |  |  | 0.79 | 0.82 | 0.82 | 0.83 | 0.89 | 0.93 | 0.91 | 0.75 |  |  | 0.84 | 0.72 |  |
| 9* | 8492 | 23 | 22 | 16 | 10 | 10 | 7 | 7 | 5 | 1 |  | 0.82 | 0.79 | 0.79 | 0.83 | 0.80 | 0.88 | 0.90 | 0.85 | 0.98 |  | 0.85 | 0.69 |  |
| 10* | 8517 | 23 | 20 | 16 | 13 | 9 | 7 | 5 | 5 | 2 | <1 | 0.83 | 0.78 | 0.98 | 0.82 | 0.83 | 0.86 | 0.89 | 0.77 | 0.98 | >0.99 | 0.86 | 0.70 |  |

*Unstable estimates or non-convergence

**
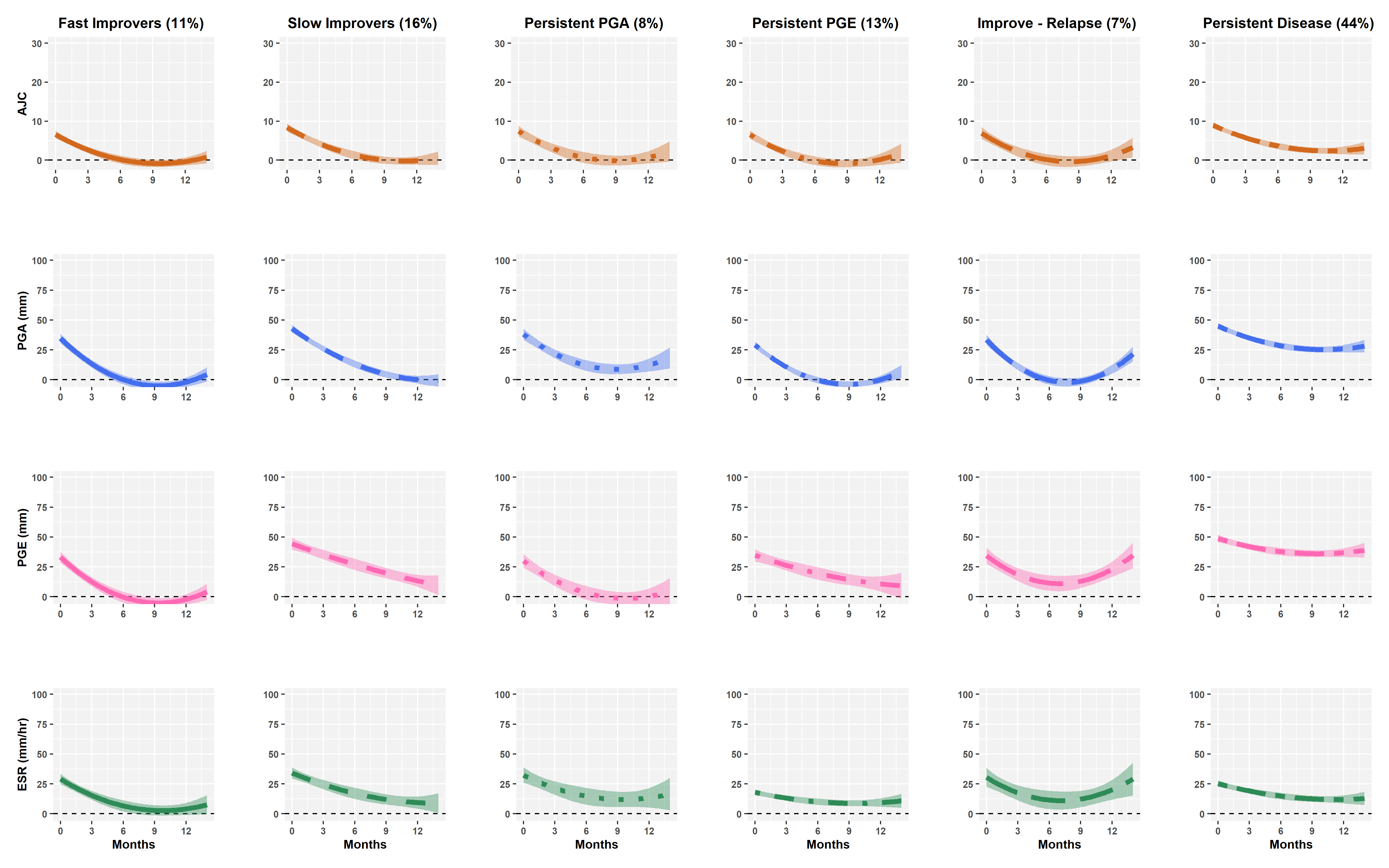
**

**Supplementary Figure 1.** Average active joint count (0-71), physician’s global (mm), patient/parent global (mm) and ESR (mm/hr) over the year following MTX initiation in the discovery cohort. Clusters were defined using log1p transformed outcomes, and are presented in raw form here to aid interpretation.

1.
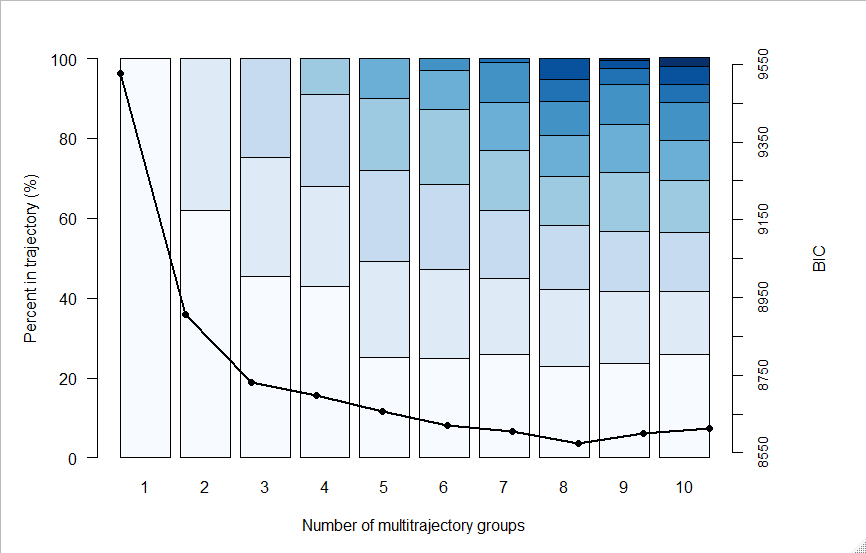

2.
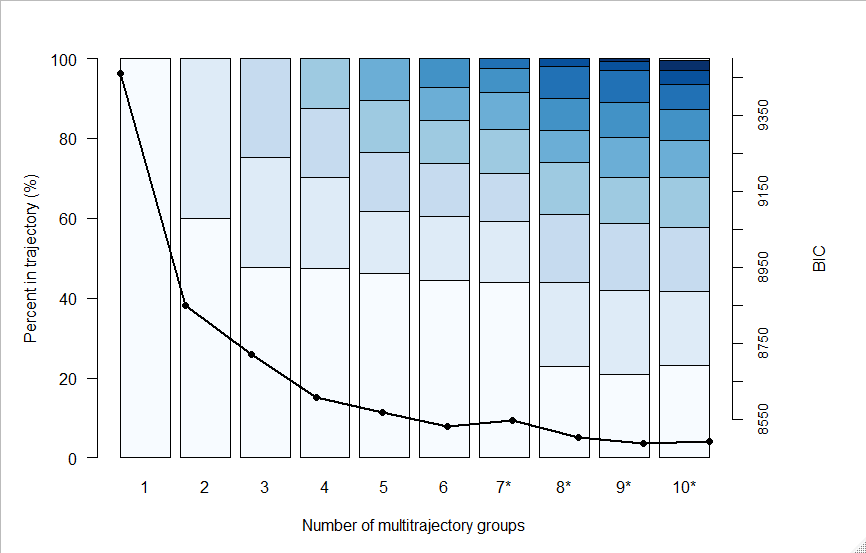

3.
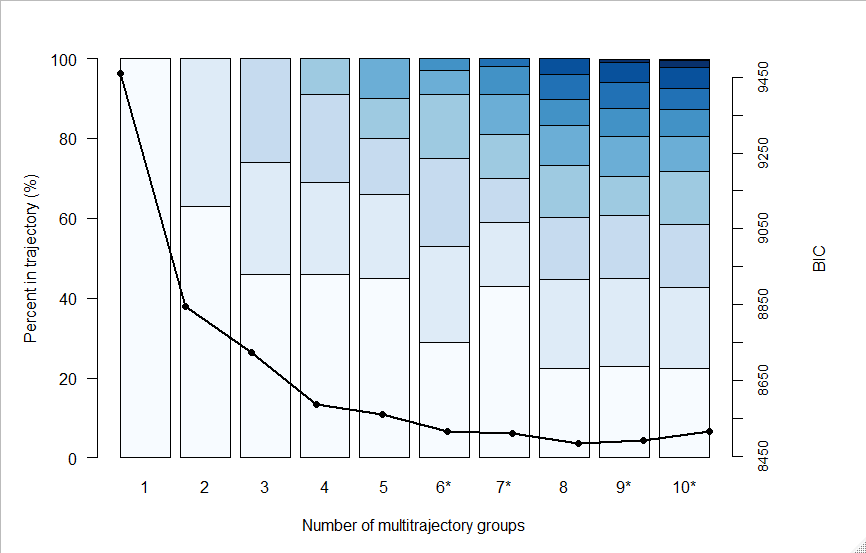


**Supplementary Figure 2.** Model fit and proportion in each class across multivariate trajectory models of JADAS component following MTX in the discovery cohort. a. Linear, b. Quadratic, c. Cubic. *Unstable estimates or non-convergence. BIC estimates have been transformed to positive values for visualisation.

**Supplementary Figure 3.** Distribution of available JADAS component data over between six clusters in the year following MTX in the discovery cohort.

**Supplementary Figure 4.** Time between registration and MTX start across clusters with different speeds of MTX response in the discovery cohort.


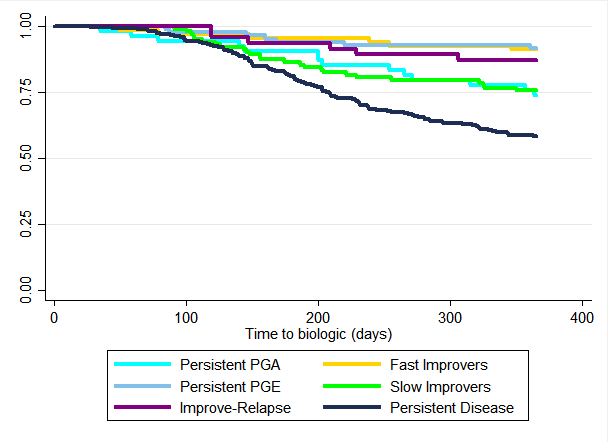


**Supplementary Figure 5.** Time to biologic therapy across the six multi-trajectory groups following MTX in the discovery cohort

**Supplementary Table 2.** ACR Pedi scores across each cluster following MTX initiation in the discovery cohort

| **ACR Pedi score** | **Percent with ACR Pedi (95% CI)** | | | | | | |
| --- | --- | --- | --- | --- | --- | --- | --- |
|  | **Total cohort** | **Trajectory group** | | | | | |
|  |  | **1**  **Pers PGA** | **2**  **Fast Imp** | **3**  **Pers PGE** | **4**  **Slow Imp** | **5**  **Imp-Rel** | **6**  **Persistent** |
| **Six months** | | | | | | | |
| ACR Pedi 30 | 74  (70, 78) | 81  (68, 94) | 92  (84, >99) | 86  (78, 94) | 73  (63, 83) | 86  (71, >99) | 62  (56, 69) |
| ACR Pedi 90 | 39  (35, 43) | 41  (25, 56) | 86  (76, 97) | 73  (62, 84) | 30  (20, 40) | 68  (62, 85) | 14  (9, 19) |
| **12 months** | | | | | | | |
| ACR Pedi 30 | 78  (74, 82) | 86  (73, 99) | 91  (81, >99) | 87  (75, 98) | 88  (81, 96) | 79  (66, 92) | 66  (59, 73) |
| ACR Pedi 90 | 50  (45, 54) | 62  (45, 79) | 86  (74, 98) | 75  (62, 87) | 73  (64, 83) | 42  (26, 57) | 23  (16, 30) |

**Supplementary Table 3.** Univariable associations between characteristics of children and young people and MTX response clusters in the discovery cohort

| **Baseline variable** | **N (%) or Median (IQR)** | | | | | | **P-value** |
| --- | --- | --- | --- | --- | --- | --- | --- |
|  | **Group 1:**  **Persistent PGA** | **Group 2:**  **Fast improvers** | **Group 3:**  **Persistent PGE** | **Group 4:**  **Slow improvers** | **Group 5:**  **Improve-Relapse** | **Group 6: Persistent disease** |  |
| **N (%)** | 54 (8) | 70 (11) | 88 (13) | 105 (16) | 48 (7) | 292 (44) |  |
| **Demographic** |  |  |  |  |  |  |  |
| Age at registration (years) | 7 (3, 11) | 9 (3, 13) | 9 (4, 12) | 7 (3, 11) | 7 (3, 12) | 10 (6, 13) | <0.001 |
| Female | 36 (68) | 44 (64) | 61 (70) | 72 (69) | 37 (77) | 198 (68) | 0.767 |
| White ethnicity | 45 (85) | 61 (87) | 70 (81) | 81 (79) | 38 (81) | 249 (86) | 0.477 |
| **ILAR category** |  |  |  |  |  |  |  |
| Systemic | 2 (4) | 1 (1) | 5 (6) | 1 (1) | 3 (7) | 8 (3) | 0.211 |
| Oligo – Pers | 9 (17) | 15 (22) | 13 (15) | 26 (26) | 4 (9) | 51 (18) |  |
| Oligo – Ext | 7 (13) | 16 (23) | 21 (24) | 14 (14) | 12 (26) | 45 (16) |  |
| RF- Poly | 22 (42) | 18 (26) | 31 (35) | 32 (32) | 15 (33) | 91 (32) |  |
| RF+ Poly | 3 (6) | 7 (10) | 4 (5) | 15 (15) | 4 (9) | 31 (11) |  |
| Psoriatic | 3 (6) | 5 (7) | 6 (7) | 5 (5) | 6 (13) | 23 (8) |  |
| Enthesitis-related | 3 (6) | 3 (4) | 3 (3) | 6 (6) | 2 (4) | 24 (9) |  |
| Undifferentiated | 3 (6) | 4 (6) | 5 (6) | 1 (1) | 0 (0) | 9 (3) |  |
| **Disease activity** |  |  |  |  |  |  |  |
| Active joint count | 5 (3, 9) | 5 (2, 8) | 4 (2, 7) | 5 (3, 11) | 5 (3, 9) | 6 (3, 12) | 0.196 |
| Limited joint count | 4 (2, 6) | 3 (2, 7) | 3 (1, 6) | 3 (1, 8) | 3 (2, 5) | 4 (2, 9) | 0.022 |
| Physician’s global (cm) | 3.9 (2.4, 5.0) | 3.0 (2.0, 6.0) | 2.8 (1.5, 4.0) | 4.0 (2.5, 6.0) | 3.0 (2.3, 5.0) | 4.2 (2.9, 6.2) | <0.001 |
| Parental global (cm) | 2.7 (0.5, 5.0) | 3.5 (1.0, 6.0) | 3.8 (1.0, 5.5) | 4.7 (2.4, 6.6) | 2.8 (1.2, 5.9) | 5.0 (3.3, 7.0) | <0.001 |
| CHAQ | 0.88 (0.13, 1.75) | 0.75 (0.13, 1.25) | 0.88 (0.50, 1.38) | 1.13 (0.50, 1.75) | 0.88 (0.25, 1.38) | 1.13 (0.50, 1.75) | 0.023 |
| ESR | 22 (8, 49) | 22 (8, 41) | 12 (7, 27) | 28 (10, 45) | 26 (10, 35) | 15 (7, 31) | 0.003 |
| Pain | 3.5 (1.0, 5.9) | 2.8 (1.0, 6.0) | 4.3 (1.6, 6.2) | 5.6 (2.8, 8.0) | 3.0 (1.5, 5.6) | 5.3 (3.1, 7.1) | <0.001 |
| History of uveitis (%)  (subset n=603) | 6 (12) | 7 (11) | 9 (11) | 12 (12) | 3 (7) | 16 (6) | 0.331 |
| Current uveitis (%) (subset of yes for history) | 5 (83) | 4 (57) | 8 (89) | 10 (77) | 1 (33) | 14 (88) | 0.237 |
| **Medication** |  |  |  |  |  |  |  |
| Ever IA steroids within one year (%)  (available data n=639) | 27 (51) | 15 (21) | 30 (34) | 45 (44) | 19 (40) | 128 (46) | 0.003 |
| Ever IV or IM steroids within one year (%)  (available data n=639) | 6 (11) | 4 (6) | 2 (2) | 9 (9) | 2 (4) | 42 (15) | 0.004 |
| Ever biologic within one year (%) | 13 (24) | 6 (9) | 7 (8) | 23 (22) | 6 (13) | 111 (38) | <0.001 |
| Intolerance/adverse event as reason to stop MTX (%)* | 3 (6) | 12 (17) | 14 (16) | 11 (10) | 6 (13) | 38 (13) | 0.414 |

**Supplementary Table 4.** Multivariable associations between characteristics of children and young people with MTX response clusters in the discovery cohort following multiple imputation of missing data

| **Baseline variable** | **OR (95% CI)** | | | | | |
| --- | --- | --- | --- | --- | --- | --- |
|  | **Group 1:**  **Persistent PGA** | **Group 2:**  **Fast improvers** | **Group 3:**  **Persistent PGE** | **Group 4:**  **Slow improvers** | **Group 5:**  **Improve-Relapse** | **Group 6: Persistent disease** |
| **N (%)** | 54 (8) | 70 (11) | 88 (13) | 105 (16) | 48 (7) | 292 (44) |
| **Demographic** |  |  |  |  |  |  |
| Age at registration (years) | 0.93 (0.86, 1.01) | **0.92 (0.86, 1.00)** | 0.95 (0.88, 1.02) | **0.89 (0.83, 0.96)** | **0.91 (0.83, 1.00)** | Reference |
| Disease duration to registration (~MTX initiation) (years) | 0.93 (0.72, 1.19) | 1.10 (0.88, 1.37) | 1.03 (0.76, 1.42) | 1.13 (0.88, 1.44) | 1.04 (0.72, 1.51) | Reference |
| Female | 0.93 (0.45, 1.91) | 0.73 (0.38, 1.42) | 1.03 (0.55, 1.93) | 0.88 (0.46, 1.69) | 1.19 (0.50, 2.81) | Reference |
| White ethnicity | 0.78 (0.32, 1.90) | 1.14 (0.44, 2.95) | **0.46 (0.21, 1.00)** | 0.69 (0.34, 1.40) | 0.52 (0.20, 1.36) | Reference |
| **ILAR category** |  |  |  |  |  |  |
| Systemic | 0.97 (0.14, 6.62) | 0.35 (0.03, 3.82) | 5.32 (0.86, 33.00) | 0.08 (0.01, 1.16) | 4.70 (0.62, 35.43) | Reference |
| Oligo – Pers | Reference | Reference | Reference | Reference | Reference | Reference |
| Oligo – Ext | 0.71 (0.22, 2.31) | 1.22 (0.48, 3.07) | 2.42 (0.96, 6.10) | 0.42 (0.18, 1.05) | 3.24 (0.87, 12.13) | Reference |
| RF- Poly | 1.52 (0.57, 4.04) | 1.00 (0.38, 2.61) | 2.39 (0.89, 6.39) | 0.45 (0.19, 1.10) | 2.48 (0.66, 9.39) | Reference |
| RF+ Poly | 0.96 (0.20, 4.65) | 1.91 (0.48, 7.56) | 1.11 (0.20, 6.20) | 0.88 (0.27, 2.93) | 2.92 (0.48, 17.67) | Reference |
| Psoriatic | 1.37 (0.21, 4.79) | 1.29 (0.34, 4.95) | 2.54 (0.68, 9.46) | 0.51 (0.13, 1.96) | 5.08 (1.03, 25.13) | Reference |
| Enthesitis-related | 1.01 (0.21, 4.79) | 0.52 (0.11, 2.43) | 0.93 (0.19, 4.55) | 0.76 (0.21, 2.83) | **<0.01 (<0.01, 0.01)** | Reference |
| Undifferentiated | 1.51 (0.72, 1.19) | 1.41 (0.27, 7.31) | 3.96 (0.89, 17.57) | 0.20 (0.02, 2.07) | **<0.01 (<0.01, 0.44)** | Reference |
| **Disease activity** |  |  |  |  |  |  |
| Active joint count | 0.99 (0.95, 1.04) | 1.00 (0.94, 1.05) | 1.00 (0.94, 1.06) | 1.02 (0.96, 1.08) | 1.00 (0.94, 1.06) | Reference |
| Limited joint count  (excluded due to collinearity with AJC) | - | - | - | - | - | Reference |
| Physician’s global (mm) | 1.00 (0.98, 1.02) | 1.00 (0.96, 1.04) | 0.97 (0.92, 1.02) | 1.00 (0.96, 1.04) | 0.99 (0.93, 1.05) | Reference |
| Parental global (mm) | **0.97 (0.96, 0.99)** | 0.99 (0.96, 1.02) | 0.99 (0.96, 1.03) | 0.99 (0.97, 1.02) | 0.98 (0.95, 1.02) | Reference |
| CHAQ | 0.98 (0.56, 1.69) | 0.48 (0.19, 1.20) | 0.97 (0.32, 2.74) | 0.95 (0.27, 3.26) | 0.98 (0.29, 3.36) | Reference |
| ESR | 1.01 (1.00, 1.02) | 1.01 (1.00, 1.03) | **0.98 (0.97, 1.00)** | 1.01 (1.00, 1.03) | 1.01 (0.99, 1.02) | Reference |
| Pain  (excluded due to collinearity with parent global) | - | - | - | - | - | Reference |
| History of uveitis | 2.26 (0.76, 6.69) | 1.30 (0.45, 3.79) | 1.58 (0.57, 4.34) | 1.86 (0.74, 4.73) | 0.91 (0.22, 3.70) |  |


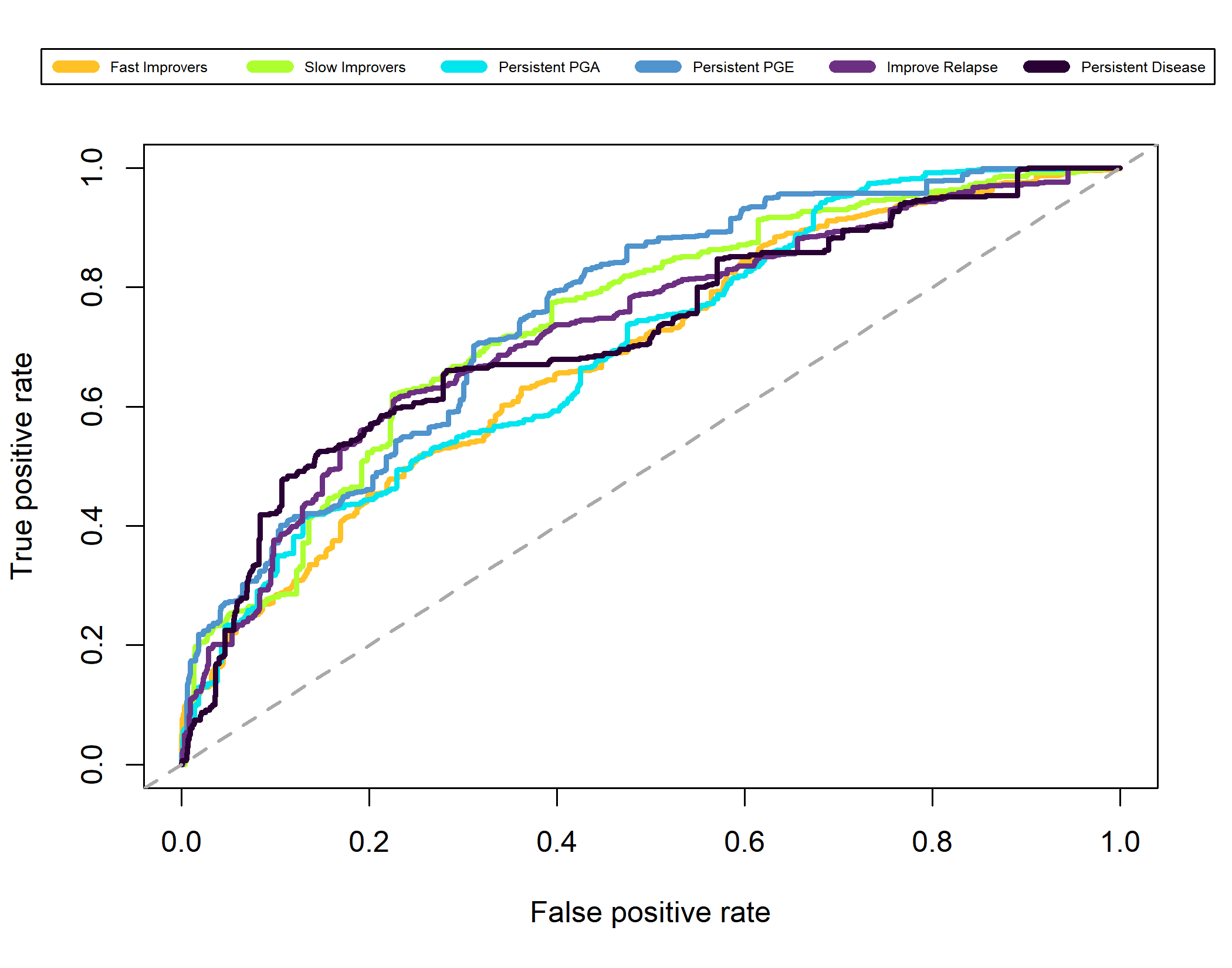


**Supplementary Figure 6.** Area under the curve values for predicting cluster of interest compared with all other clusters.

**Supplementary Table 5.** Model diagnostics across multi-trajectory models developed in the CAPS verification cohort (n=575)

| Class no. | BIC | Class percentages (%) | | | | | | | | | | Average posterior probability per class  (higher is better) | | | | | | | | | | Model average posterior prob. for assigned class  (higher is better) | Relative Entropy  (close to 1 is better) | |
| --- | --- | --- | --- | --- | --- | --- | --- | --- | --- | --- | --- | --- | --- | --- | --- | --- | --- | --- | --- | --- | --- | --- | --- | --- |
|  |  | 1 | 2 | 3 | 4 | 5 | 6 | 7 | 8 | 9 | 10 | 1 | 2 | 3 | 4 | 5 | 6 | 7 | 8 | 9 | 10 |  |  |  |
| Linear | | | | | | | | | | | | | | | | | | | | | | | | |
| 1 | -7748.49 | 1 |  |  |  |  |  |  |  |  |  | 1 |  |  |  |  |  |  |  |  |  | 1 | 1 |  |
| 2 | -7474.5 | 0.63 | 0.37 |  |  |  |  |  |  |  |  | 0.94 | 0.89 |  |  |  |  |  |  |  |  | 0.913882 | 0.884894 |  |
| 3 | -7421.19 | 0.58 | 0.24 | 0.18 |  |  |  |  |  |  |  | 0.93 | 0.83 | 0.85 |  |  |  |  |  |  |  | 0.87186 | 0.83882 |  |
| 4 | -7384.93 | 0.50 | 0.22 | 0.18 | 0.10 |  |  |  |  |  |  | 0.90 | 0.79 | 0.82 | 0.86 |  |  |  |  |  |  | 0.844128 | 0.782855 |  |
| 5 | -7373.48 | 0.28 | 0.27 | 0.17 | 0.14 | 0.13 |  |  |  |  |  | 0.77 | 0.80 | 0.80 | 0.85 | 0.81 |  |  |  |  |  | 0.809698 | 0.689389 |  |
| 6 | -7357.92 | 0.27 | 0.27 | 0.16 | 0.14 | 0.10 | 0.05 |  |  |  |  | 0.80 | 0.78 | 0.80 | 0.80 | 0.83 | 0.80 |  |  |  |  | 0.802538 | 0.682382 |  |
| 7 | -7356.21 | 0.27 | 0.25 | 0.16 | 0.14 | 0.09 | 0.05 | 0.04 |  |  |  | 0.79 | 0.79 | 0.81 | 0.81 | 0.81 | 0.79 | 0.78 |  |  |  | 0.79581 | 0.67711 |  |
| 8 | -7363.69 | 0.28 | 0.24 | 0.15 | 0.13 | 0.09 | 0.06 | 0.04 | 0.02 |  |  | 0.80 | 0.77 | 0.82 | 0.81 | 0.82 | 0.75 | 0.76 | 0.80 |  |  | 0.790863 | 0.669715 |  |
| 9 | -7371.74 | 0.27 | 0.24 | 0.15 | 0.13 | 0.08 | 0.06 | 0.04 | 0.02 | 0.01 |  | 0.79 | 0.77 | 0.81 | 0.81 | 0.79 | 0.77 | 0.79 | 0.80 | 0.91 |  | 0.804907 | 0.663465 |  |
| 10 | -7377.65 | 0.25 | 0.22 | 0.10 | 0.09 | 0.08 | 0.08 | 0.07 | 0.07 | 0.04 | 0.01 | 0.76 | 0.79 | 0.76 | 0.76 | 0.78 | 0.80 | 0.72 | 0.84 | 0.79 | 0.96 | 0.796776 | 0.626835 |  |
| Quadratic | | | | | | | | | | | | | | | | | | | | | | | | |
| 1 | -7662.36 | 1 |  |  |  |  |  |  |  |  |  | 1 |  |  |  |  |  |  |  |  |  | 1 | 1 |  |
| 2 | -7411.61 | 0.64 | 0.36 |  |  |  |  |  |  |  |  | 0.93 | 0.90 |  |  |  |  |  |  |  |  | 0.912322 | 0.877687 |  |
| 3 | -7375.37 | 0.44 | 0.37 | 0.19 |  |  |  |  |  |  |  | 0.87 | 0.80 | 0.90 |  |  |  |  |  |  |  | 0.858696 | 0.777903 |  |
| 4 | -7351.82 | 0.39 | 0.31 | 0.17 | 0.13 |  |  |  |  |  |  | 0.86 | 0.79 | 0.81 | 0.88 |  |  |  |  |  |  | 0.831704 | 0.735586 |  |
| 5 | -7362.91 | 0.38 | 0.30 | 0.13 | 0.12 | 0.07 |  |  |  |  |  | 0.86 | 0.79 | 0.84 | 0.84 | 0.80 |  |  |  |  |  | 0.826268 | 0.723074 |  |
| 6 | -7334.23 | 0.32 | 0.19 | 0.16 | 0.14 | 0.12 | 0.07 |  |  |  |  | 0.82 | 0.75 | 0.80 | 0.84 | 0.79 | 0.88 |  |  |  |  | 0.81243 | 0.690962 |  |
| 7* | -7334.57 | 0.29 | 0.23 | 0..15 | 0.14 | 0.07 | 0.06 | 0.05 |  |  |  | 0.82 | 0.78 | 0.78 | 0.80 | 0.83 | 0.83 | 0.84 |  |  |  | 0.813372 | 0.686739 |  |
| 8* | -7364.32 | 0.27 | 0.25 | 0.16 | 0.12 | 0.06 | 0.06 | 0.05 | 0.02 |  |  | 0.79 | 0.80 | 0.77 | 0.84 | 0.81 | 0.80 | 0.81 | 0.89 |  |  | 0.813361 | 0.679207 |  |
| 9* | -7363.63 | 0.26 | 0.25 | 0.16 | 0.07 | 0.07 | 0.05 | 0.05 | 0.05 | 0.03 |  | 0.82 | 0.79 | 0.81 | 0.78 | 0.81 | 0.87 | 0.84 | 0.87 | 0.80 |  | 0.822235 | 0.692318 |  |
| 10* | -7383.05 | 0.26 | 0.25 | 0.15 | 0.07 | 0.07 | 0.06 | 0.05 | 0.04 | 0.04 | 0.02 | 0.82 | 0.79 | 0.78 | 0.84 | 0.81 | 0.86 | 0.83 | 0.88 | 0.81 | 0.82 | 0.824591 | 0.679893 |  |
| Cubic | | | | | | | | | | | | | | | | | | | | | | | | |
| 1 | -7650.01 | 1 |  |  |  |  |  |  |  |  |  | 1 |  |  |  |  |  |  |  |  |  | 1 | 1 |  |
| 2 | -7397.67 | 0.65 | 0.35 |  |  |  |  |  |  |  |  | 0.93 | 0.91 |  |  |  |  |  |  |  |  | 0.921199 | 0.890573 |  |
| 3 | -7374.28 | 0.52 | 0.33 | 0.16 |  |  |  |  |  |  |  | 0.89 | 0.81 | 0.90 |  |  |  |  |  |  |  | 0.867292 | 0.80133 |  |
| 4 | -7359.85 | 0.48 | 0.22 | 0.19 | 0.11 |  |  |  |  |  |  | 0.88 | 0.80 | 0.82 | 0.86 |  |  |  |  |  |  | 0.839528 | 0.767213 |  |
| 5 | -7379.06 | 0.47 | 0.19 | 0.18 | 0.09 | 0.08 |  |  |  |  |  | 0.90 | 0.79 | 0.83 | 0.83 | 0.90 |  |  |  |  |  | 0.850772 | 0.770492 |  |
| 6 | -7391.97 | 0.44 | 0.19 | 0.11 | 0.10 | 0.09 | 0.07 |  |  |  |  | 0.87 | 0.83 | 0.80 | 0.79 | 0.82 | 0.83 |  |  |  |  | 0.822349 | 0.726425 |  |
| 7* | -7388.56 | 0.28 | 0.26 | 0.14 | 0.13 | 0.08 | 0.07 | 0.04 |  |  |  | 0.81 | 0.80 | 0.80 | 0.84 | 0.83 | 0.81 | 0.88 |  |  |  | 0.825303 | 0.69927 |  |
| 8* | -7409.91 | 0.27 | 0.26 | 0.17 | 0.10 | 0.07 | 0.07 | 0.04 | 0.02 |  |  | 0.82 | 0.82 | 0.81 | 0.87 | 0.81 | 0.84 | 0.91 | 0.90 |  |  | 0.847526 | 0.718866 |  |
| 9* | -7441.98 | 0.26 | 0.18 | 0.13 | 0.12 | 0.10 | 0.07 | 0.07 | 0.04 | 0.03 |  | 0.78 | 0.75 | 0.79 | 0.82 | 0.78 | 0.78 | 0.88 | 0.81 | 0.87 |  | 0.805341 | 0.658163 |  |
| 10* | -7488.74 | 0.27 | 0.18 | 0.13 | 0.10 | 0.09 | 0.07 | 0.06 | 0.04 | 0.03 | 0.03 | 0.79 | 0.72 | 0.78 | 0.81 | 0.83 | 0.87 | 0.81 | 0.78 | 0.94 | 0.88 | 0.821385 | 0.657002 |  |

*Unstable estimates or non-convergence

1.
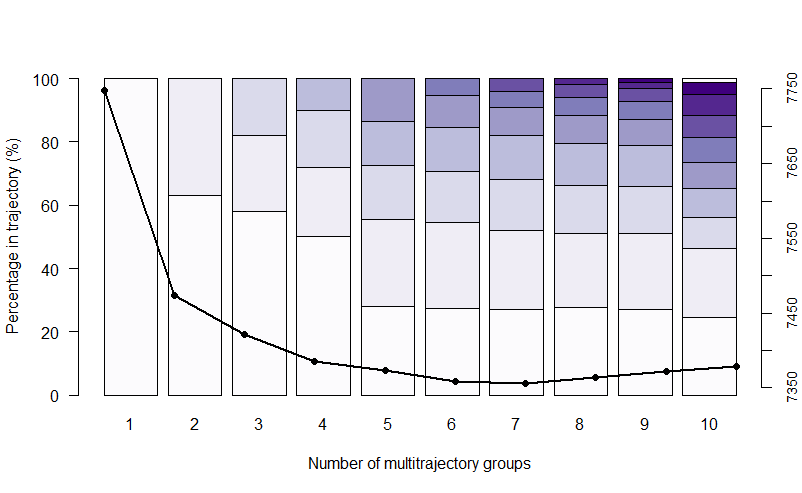

2.
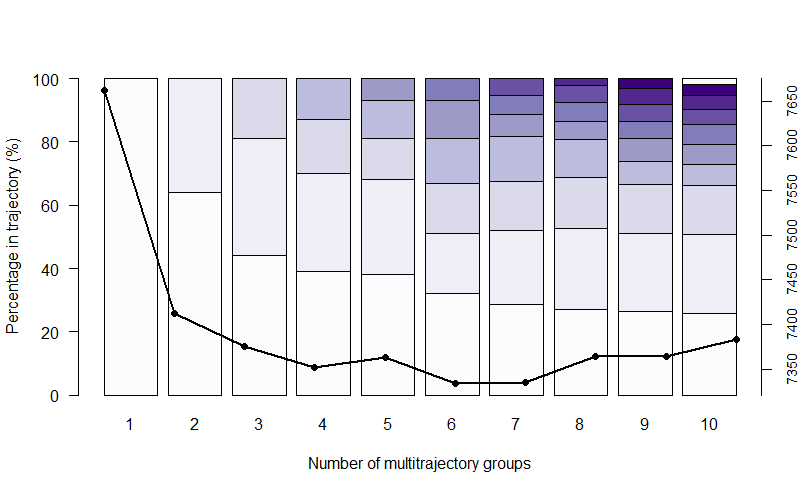

3.
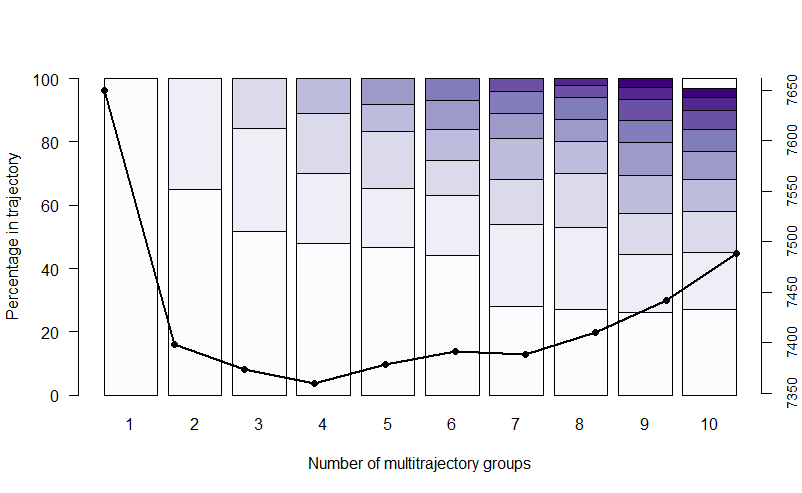


**Supplementary Figure 7.** Percent in each cluster and BIC across multi-trajectory models developed in the CAPS verification cohort (n=575): a) Linear models, b) quadratic models, c) cubic models. BIC estimates have been transformed to positive values for visualisation.

**Supplementary Table 6.** Model diagnostics across multi-trajectory models developed in the CHARMS verification cohort (n=660)

| Class no. | BIC | Class proportions | | | | | | | | | | Average posterior probability per class  (higher is better) | | | | | | | | | | Model average posterior prob. for assigned class  (higher is better) | Relative Entropy  (close to 1 is better) | |
| --- | --- | --- | --- | --- | --- | --- | --- | --- | --- | --- | --- | --- | --- | --- | --- | --- | --- | --- | --- | --- | --- | --- | --- | --- |
|  |  | 1 | 2 | 3 | 4 | 5 | 6 | 7 | 8 | 9 | 10 | 1 | 2 | 3 | 4 | 5 | 6 | 7 | 8 | 9 | 10 |  |  |  |
| Linear | | | | | | | | | | | | | | | | | | | | | | | | |
| 1 | -7247.32 | 1 |  |  |  |  |  |  |  |  |  | 1 |  |  |  |  |  |  |  |  |  | 1 | 1 |  |
| 2 | -6514.31 | 0.737879 | 0.262121 |  |  |  |  |  |  |  |  | 0.978989 | 0.957321 |  |  |  |  |  |  |  |  | 0.968155 | 0.968 |  |
| 3 | -6344.35 | 0.4 | 0.362121 | 0.237879 |  |  |  |  |  |  |  | 0.891575 | 0.875263 | 0.966768 |  |  |  |  |  |  |  | 0.911202 | 0.876 |  |
| 4 | -6319.41 | 0.312121 | 0.295455 | 0.239394 | 0.15303 |  |  |  |  |  |  | 0.861566 | 0.83229 | 0.961765 | 0.780211 |  |  |  |  |  |  | 0.858958 | 0.816 |  |
| 5 | -6223.56 | 0.392424 | 0.259091 | 0.119697 | 0.115152 | 0.113636 |  |  |  |  |  | 0.892808 | 0.820506 | 0.960614 | 0.917912 | 0.90575 |  |  |  |  |  | 0.899518 | 0.843 |  |
| 6 | -6205.33 | 0.381818 | 0.259091 | 0.115152 | 0.110606 | 0.106061 | 0.027273 |  |  |  |  | 0.887854 | 0.819983 | 0.912001 | 0.911305 | 0.919725 | 0.945627 |  |  |  |  | 0.899416 | 0.835 |  |
| 7* | -6246.14 | 0.266667 | 0.234848 | 0.14697 | 0.112121 | 0.109091 | 0.098485 | 0.031818 |  |  |  | 0.755181 | 0.835589 | 0.78694 | 0.87919 | 0.924587 | 0.863407 | 0.897512 |  |  |  | 0.848915 | 0.754 |  |
| 8 | -6150.96 | 0.248485 | 0.210606 | 0.130303 | 0.110606 | 0.107576 | 0.101515 | 0.071212 | 0.019697 |  |  | 0.846322 | 0.773612 | 0.799167 | 0.871716 | 0.869269 | 0.902082 | 0.859858 | 0.951388 |  |  | 0.859177 | 0.767 |  |
| 9 | -6144.43 | 0.209091 | 0.2 | 0.128788 | 0.112121 | 0.106061 | 0.093939 | 0.068182 | 0.063636 | 0.018182 |  | 0.76025 | 0.835895 | 0.781433 | 0.875003 | 0.873427 | 0.910468 | 0.861664 | 0.763463 | 0.990166 |  | 0.850197 | 0.751 |  |
| 10* | -6119.39 | 0.213636 | 0.186364 | 0.137879 | 0.110606 | 0.104545 | 0.077273 | 0.062121 | 0.057576 | 0.033333 | 0.016667 | 0.754353 | 0.848681 | 0.787937 | 0.86302 | 0.893752 | 0.880377 | 0.771382 | 0.865935 | 0.910299 | 0.966007 | 0.854174 | 0.750 |  |

*Non-singular


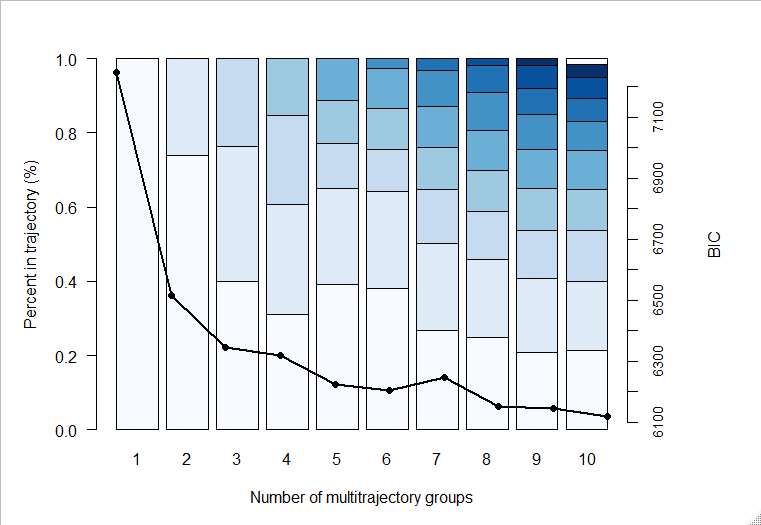


**Supplementary Figure 8.** Percent in each cluster and BIC across linear-polynomial multi-trajectory models developed in the CHARMS verification cohort. BIC estimates have been transformed to positive values for visualisation.

1.
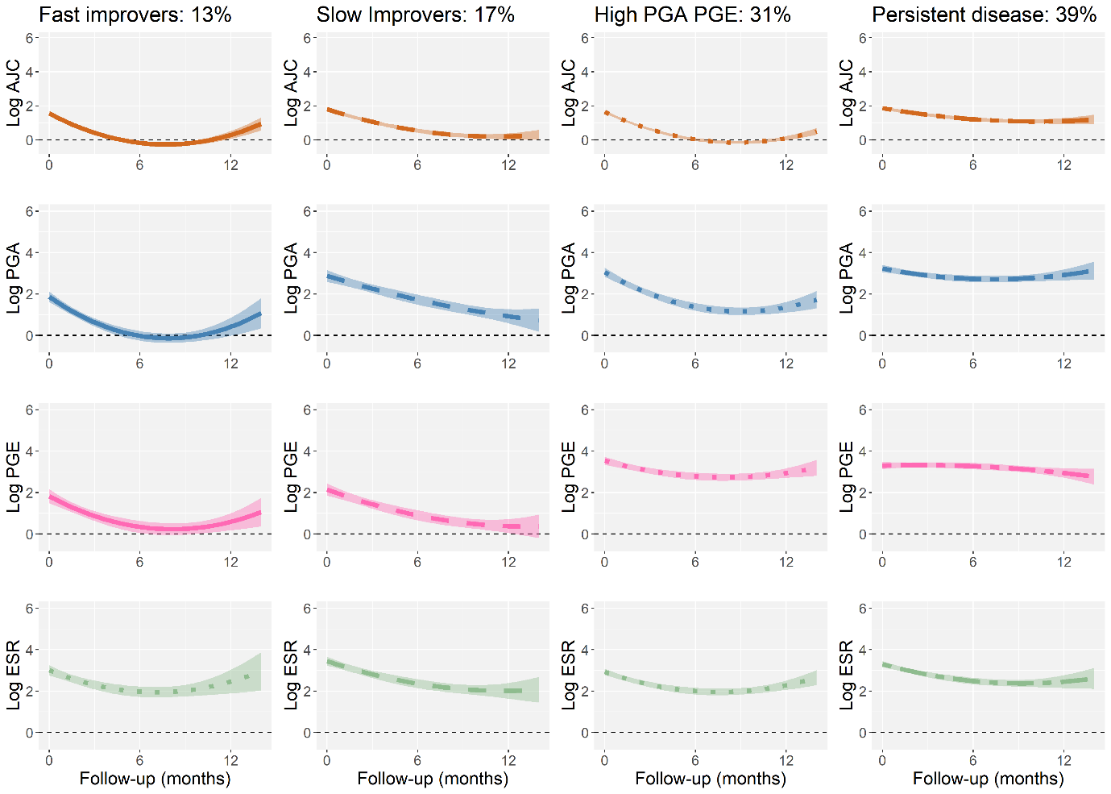

2.
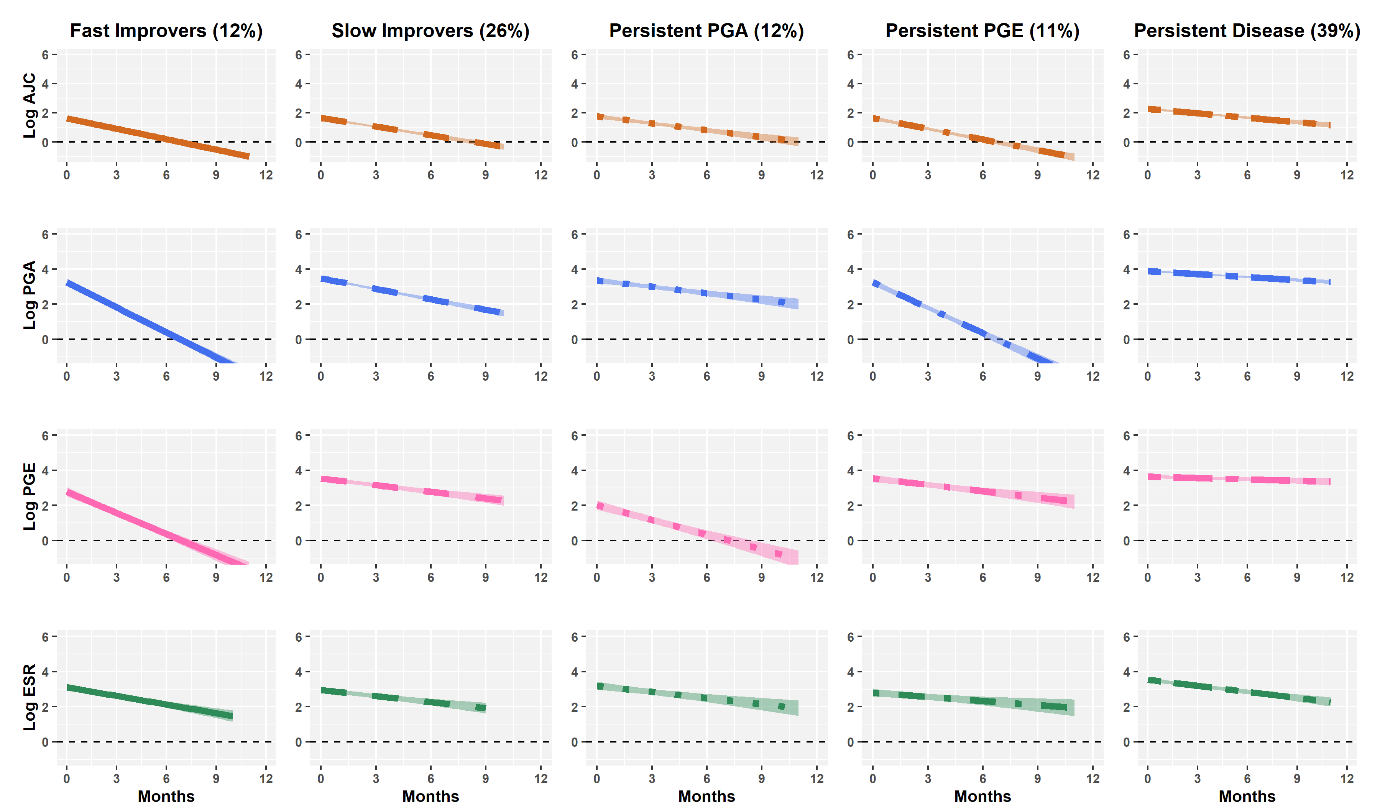


**Supplementary Figure 9**. Multivariate trajectories of JADAS components following MTX in the verification cohorts: a) CAPS, b) CHARMS
