# Supplementary material for: Towards stratified treatment of JIA: machine learning identifies subtypes in response to methotrexate from four UK cohorts
Source: eBioMedicine. 2024 Jan 8;100:104946. doi: 10.1016/j.ebiom.2023.104946 (PMC10792564; doi:10.1016/j.ebiom.2023.104946)
Supplement: Members of the CLUSTER Consortium [file mmc2.docx]

| **First names** | **Surnames** |
| --- | --- |
| Aline | Kimonyo |
| Alyssia | McNeece |
| Andrew | Dick |
| Andrew | Morris |
| Annie | Yarwood |
| Athimalaipet | Ramanan |
| Bethany R | Jebson |
| Chris | Wallace |
| Daniela | Dastros-Pitei |
| Damian | Tarasek |
| Elizabeth | Ralph |
| Emil | Carlsson |
| Emily | Robinson |
| Emma | Sumner |
| Fatema | Merali |
| Fatjon | Dekaj |
| Helen | Neale |
| Hussein | Al-Mossawi |
| Jacqui | Roberts |
| Jenna F | Gritzfeld |
| Joanna | Fairlie |
| John | Bowes |
| John | Ioannou |
| Kimme L | Hyrich |
| Lucy R | Wedderburn |
| Melissa | Kartawinata |
| Melissa | Tordoff |
| Michael | Barnes |
| Michael W | Beresford |
| Michael | Stadler |
| Nophar | Geifman |
| Paul | Martin |
| Rami | Kallala |
| Sandra | Ng |
| Samantha | Smith |
| Sarah | Clarke |
| Saskia | Lawson-Tovey |
| Soumya | Raychaudhuri |
| Stephanie JW | Shoop-Worrall |
| Stephen | Eyre |
| Sumanta | Mukherjee |
| Teresa | Duerr |
| Thierry | Sornasse |
| Vasiliki | Alexiou |
| Victoria J | Burton |
| Wei-Yu | Lin |
| Wendy | Thomson |
| Zoe | Wanstall |
